# A Structural Mimic of Carbonic Anhydrase in Zeolitic Imidazolate Frameworks via Trans-functionalization for Enhancing Hydrolytic Activity

**DOI:** 10.34133/research.0434

**Published:** 2024-08-09

**Authors:** Fanchen Meng, Cheng Xu, Linghai Zhang, Xiaohan Huang, Xinglong Zhang, Wenlei Zhang, Yongqi Luo, Weina Zhang, Wei Huang, Fengwei Huo, Suoying Zhang

**Affiliations:** Key Laboratory of Flexible Electronics (KLOFE), Institute of Advanced Materials (IAM) & School of Flexible Electronics (Future Technologies), Nanjing Tech University, Nanjing 211816, P.R. China.

## Abstract

Metal-organic frameworks (MOFs) have been widely considered as ideal platforms for the preparation of biomimetic catalysts, but it remains challenging to fabricate MOF-based enzyme-like catalysts with optimal activity. Here, we leverage the inherent flexibility of MOFs and propose a novel trans-functionalization strategy to construct a carbonic anhydrase (CA) mimic by the structural transformation from ZIF-L to ZIF-8. Theoretical and experimental results reveal that during the structural transformation, the hydroxyl group will preferentially coordinate with the interlayer Zn clusters to form the CA-like active center Zn-N_3_-OH. Therefore, more accessible active centers are generated on the as-prepared ZIF-8-OH, resulting in substantially enhanced catalytic activity in the hydrolysis of *para*-nitrophenyl acetate.

## Introduction

Enzymes are natural catalysts that exhibit high efficiency and specificity, allowing for catalyzing various complex substrates with excellence [1–3]. However, their widespread application is hindered by issues such as poor operational stability and challenges in recovery and recycling [4–8]. Biomimetic catalysts, designed to mimic key properties of enzymes, offer promising solutions to overcome these limitations [9–11]. Metal-organic frameworks (MOFs), which are constructed by metal ions/clusters and organic ligands via coordination bonds, have emerged as an ideal platform for the fabrication of biomimetic catalysts, particularly for metalloenzymes [12–15]. The isolated metal nodes, structural diversity, and tunable pore environments in MOFs enable them to mimic both the coordination structure of the metalloenzyme and the channel’s microenvironment found in the enzyme [16–18]. Therefore, in recent years, the development of enzyme mimics based on MOFs has attracted increasing attention in catalytic chemistry [19–23].

Carbonic anhydrase (CA), a zinc metalloenzyme, plays a vital role in catalyzing the interconversion between CO_2_ and bicarbonate. Its active center comprises one Zn atom coordinated by 3 imidazole N from 3 histidines and one oxygen from the hydroxyl group (Zn-N_3_-OH) [24–26]. CA is ubiquitously found in animals, plants, and microorganisms, serving essential functions in numerous areas, such as CO_2_ capture, biological detection, drug development, and biosensors [27–31]. Given its significance, many CA mimics have been developed based on MOFs [32,33]. Typically, MOFs with Zn active centers are post-functionalized through ligand exchange to generate CA-like active centers [34,35]. However, it is worth noting that these grafted active centers are predominantly located on the outer surface of MOFs, leading to the numbered active sites and suboptimal activity of CA mimics [33,36]. Thus, despite the progress, it is still highly desirable to develop a novel strategy to fabricate MOF-based CA-like catalysts with enhanced catalytic activity.

**Fig. 1.**
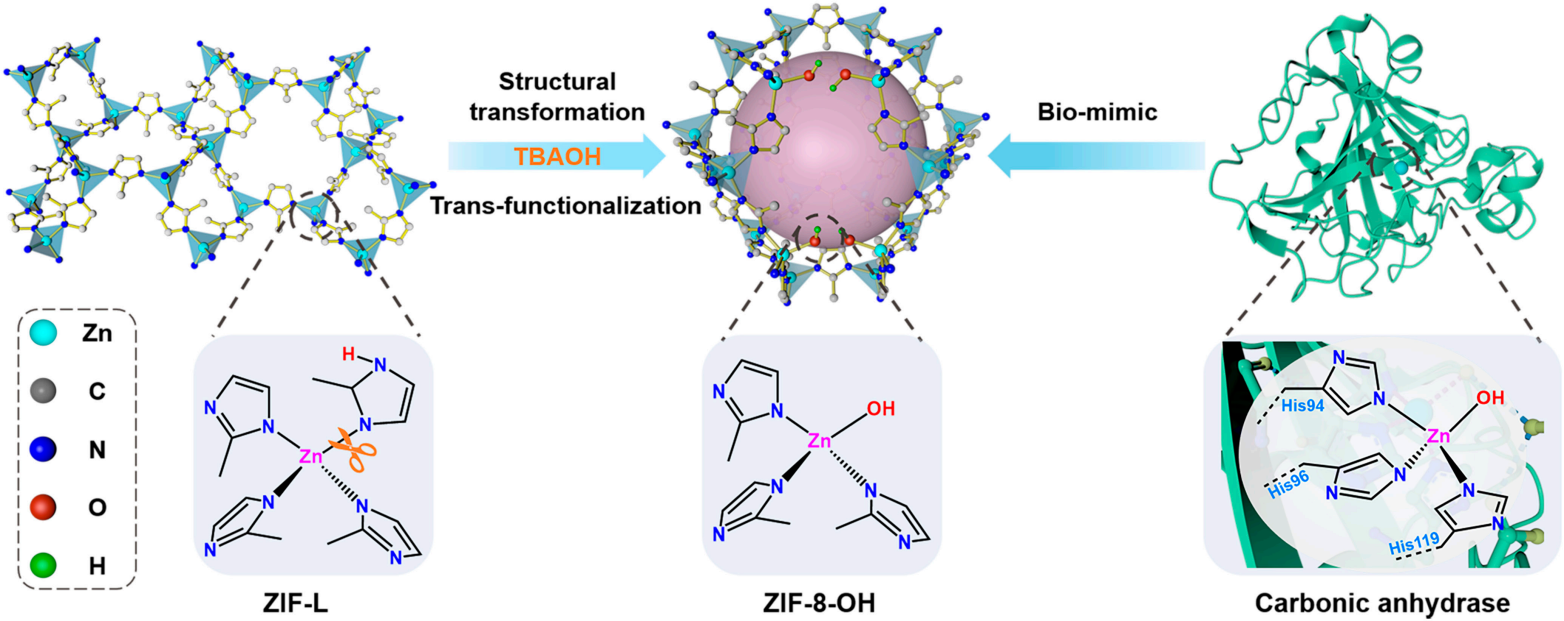
The carbonic anhydrase mimic (ZIF-8-OH) constructed by trans-functionalization during the structural transformation from ZIF-L to ZIF-8.

Structural flexibility is a key property of MOFs, enabling the breakage and recombination of coordination bonds under external stimuli to generate new MOFs [37,38]. Leveraging this process of structural transformation, we propose a novel strategy termed trans-functionalization for modifying the framework of Zn-based MOFs during structural transformation. In this strategy, tetrabutylammonium hydroxide (TBAOH) serves as the OH^−^ source to trigger the transformation from ZIF-L to ZIF-8 and competes with imidazole N to coordinate with Zn, thereby constructing CA-mimicking active centers (Fig. 1). Theoretical calculations elucidate that, compared to traditional post-functionalization methods, trans-functionalization exhibits a lower reaction energy barrier, allowing for the modification of more hydroxyl groups onto MOFs. Additionally, the grafted hydroxyl groups induce the formation of defects, leading to a hierarchical porous structure in MOFs and increased exposure of active centers. Consequently, the resulting ZIF-8-OH exhibits significantly enhanced CA-like activity in the hydrolysis of *para*-nitrophenyl acetate (*p*-NPA).

## Results

### Synthesis of CA mimics

**Fig. 2.**
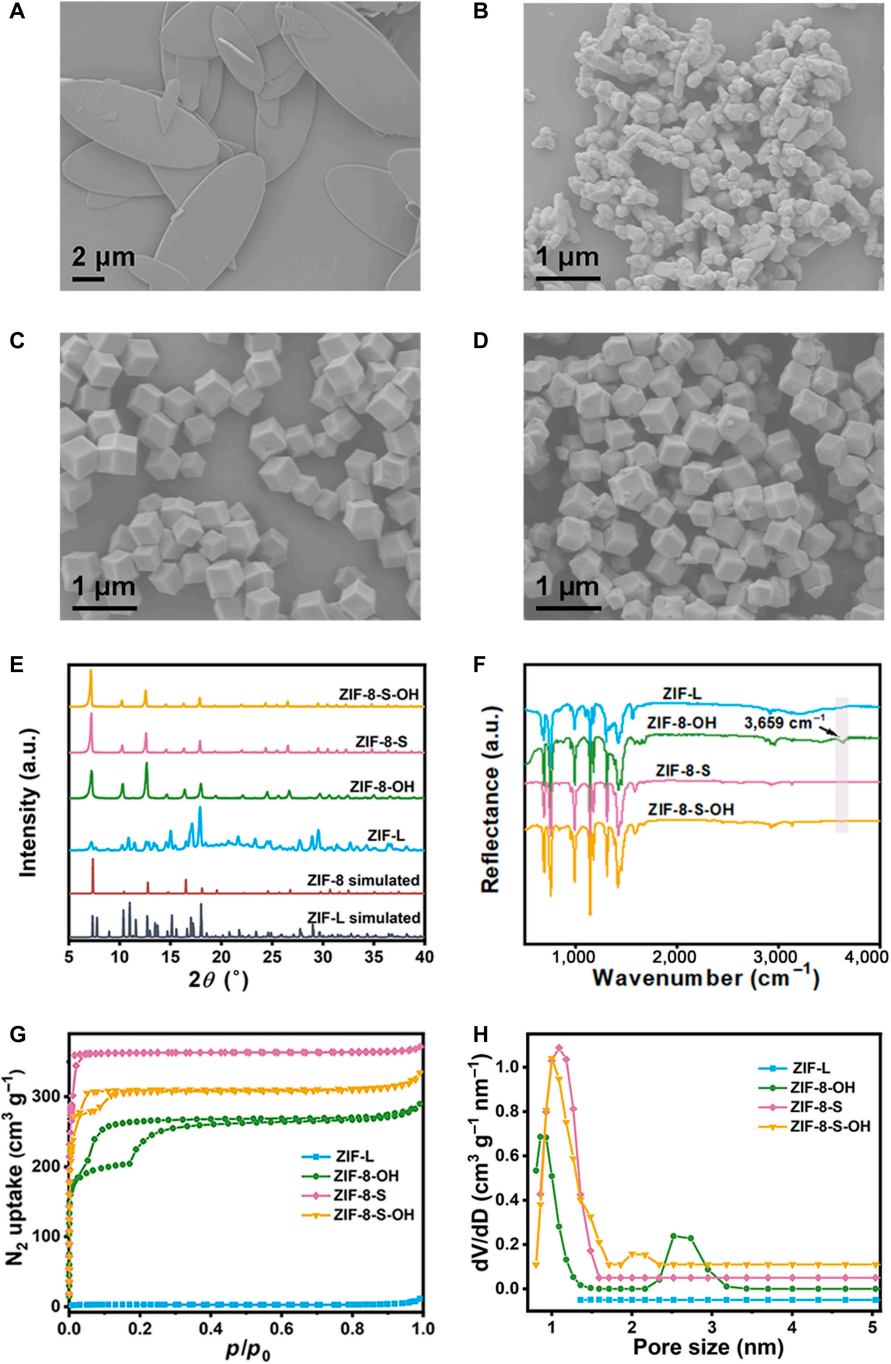
Morphology and structural characterization of samples. FESEM images of (A) pristine ZIF-L, (B) ZIF-8-OH, (C) ZIF-8-S, and (D) ZIF-8-S-OH. (E) PXRD patterns, (F) DRIFTS spectra, (G) nitrogen adsorption–desorption isotherms, and (H) pore size distributions of ZIF-L, ZIF-8-OH, ZIF-8-S, and ZIF-8-S-OH.

ZIF-L was synthesized according to the literature method [39], and ZIF-8-OH was obtained by the structural transformation of ZIF-L as described in Materials and Methods. The morphology and crystal structure of products were characterized using field emission scanning electron microscopy (FESEM) and powder x-ray diffraction (PXRD). As shown in Fig. 2A and E, ZIF-L exhibited a typical leaf-like morphology and matched well with the diffraction peaks of ZIF-L simulation. After reacting with TBAOH for 12 h, the obtained ZIF-8-OH presented the granular structure and was identified to be ZIF-8, indicating the successful structural transformation from ZIF-L to ZIF-8 (Fig. 2E). The evolution of ZIF-L morphology with different reaction times was monitored (Fig. S1A to F), showing the gradual transition from ZIF-L nanosheets to rod-like particles. According to the PXRD patterns (Fig. S2), characteristic planes of ZIF-8 (321 and 510) appeared at the initial stage, indicating that TBAOH could remove the proton in ZIF-L to trigger the structural transformation from ZIF-L to ZIF-8. With the prolonged reaction time, the intensity of ZIF-L peaks weakened, while those of ZIF-8 increased. By 8 h, characteristic peaks of ZIF-L were barely discernible. Finally, the leaf-like ZIF-L completely transformed into ZIF-8 after 10 h.

### Characterization of CA mimics

To further elucidate the role of TBAOH in the transformation of ZIF-L into ZIF-8-OH, the structural transformation of ZIF-L was also conducted in the absence of OH^−^ to obtain ZIF-8-L, following a previously reported method developed by our group [40]. Additionally, ZIF-8 synthesized conventionally (denoted as ZIF-8-S) was post-functionalized by TBAOH to obtain ZIF-8-S-OH as a comparison. Unlike ZIF-8-OH, ZIF-8-L inherits the morphology of ZIF-L (Fig. S3) while exhibiting the same long-range ordered structure as ZIF-8, as confirmed by PXRD (Fig. S4). Compared to ZIF-8-S, ZIF-8-S-OH maintained the dodecahedral architecture with a similar average diameter of about 500 nm (Fig. 2C and D), indicating the negligible effects of functionalization on the structure of ZIF-8. Interestingly, the position of (110), (200), and (211) facets in the PXRD pattern of ZIF-8-OH and ZIF-8-S-OH shifted to the left of ZIF-8-L (Fig. S4), illustrating the lattice expansion after TBAOH modification. This observation suggests that the substitution of hydroxyl groups for part of the imidazole N to form Zn-N_3_-OH induces defects in the ZIF-8 framework, leading to lattice loosening and expansion. Thus, successful introduction of the hydroxyl group in ZIF-8-OH and ZIF-8-S-OH is implied.

To confirm the formation of the Zn-N_3_-OH bonds in the products, diffuse reflectance infrared Fourier transform spectroscopy (DRIFTS) was collected as shown in Fig. 2F. ZIF-8-OH displayed a characteristic peak located at 3,659 cm^−1^ attributed to the stretching of Zn-OH. In contrast, although ZIF-8-S-OH also exhibited a peak around 3,659 cm^−1^, the peak intensity was significantly lower than ZIF-8-OH, implying a lower degree of hydroxyl group grafting. Moreover, since Zn-OH bonds were absent in ZIF-L and OH^−^ was not involved during the preparation of ZIF-8-L, no characteristic bending signals of Zn-OH were observed in the DRIFTS of ZIF-L and ZIF-8-L (Fig. 2F and Fig. S5). The atomic ratio of N to Zn in the as-prepared samples was determined using a commercial carbon, nitrogen, hydrogen, sulfur (CHNS) analyzer and inductively coupled plasma mass spectrometry. As shown in Table, the N/Zn values were 3.04, 4.54, 3.88, and 3.67, respectively. Theoretically, the ratio of Zn to N in ZIF-8 is 4:1. The notably lower values observed for ZIF-8-OH and ZIF-8-S-OH confirmed the partial replacement of Zn-N bonds by Zn-O bonds in these samples. The closer value of 3.04 in ZIF-8-OH to the theoretical ratio of 3.00 for Zn-N_3_-OH suggests a higher concentration of active sites in ZIF-8-OH compared to ZIF-8-S-OH. The unusual Zn/N ratio in ZIF-8-L could be attributed to residual dimethylformamide (DMF) since the transformation was carried out in a solvent mixture of DMF and ethanol. N_2_ adsorption–desorption isotherms were detected to evaluate the changes in pore structure (Fig. 2G and Fig. S6A). ZIF-L, being nonporous, exhibited a low specific surface area of 12 m^2^/g. After the structural transformation, both ZIF-8-OH and ZIF-8-L exhibited a porous structure. However, ZIF-8-L had a typical I sorption isotherm similar to the reported ZIF-8, while ZIF-8-OH exhibited an unusual adsorption curve with a hysteresis loop centered at *p*/*p*_0_ = 0.1. Although the hysteresis loop appeared frequently in MOFs with the hierarchical pore structure, they usually center above *p*/*p*_0_ = 0.4. The hysteresis loop located at low pressure is often associated with hydroxyl groups and defects [41], representing the strong adsorption with preferential high-energy sites. ZIF-8-S-OH displayed a smaller hysteresis loop size than ZIF-8-OH at the same pressure range, indicating fewer Zn-OH strong adsorption sites, consistent with the results above. Pore size distributions were calculated by the density functional theory (DFT) method (Fig. 2H and Fig. S6B). Micropores and mesopores were found to coexist in ZIF-8-OH and ZIF-8-S-OH, while ZIF-8-L exhibited the dominant microporous structure. The generation of active centers resulted in defect formation, leading to the appearance of mesopores in ZIF-8-OH and ZIF-8-S-OH.

**Table.** Element analysis of as-prepared different MOFs

Sample	Element (mol %)		N/Zn
	Zn	N	
ZIF-8-OH	0.46	1.4	3.04
ZIF-8-L	0.37	1.68	4.54
ZIF-8-S	0.42	1.63	3.88
ZIF-8-S-OH	0.45	1.65	3.67

**Fig. 3.**
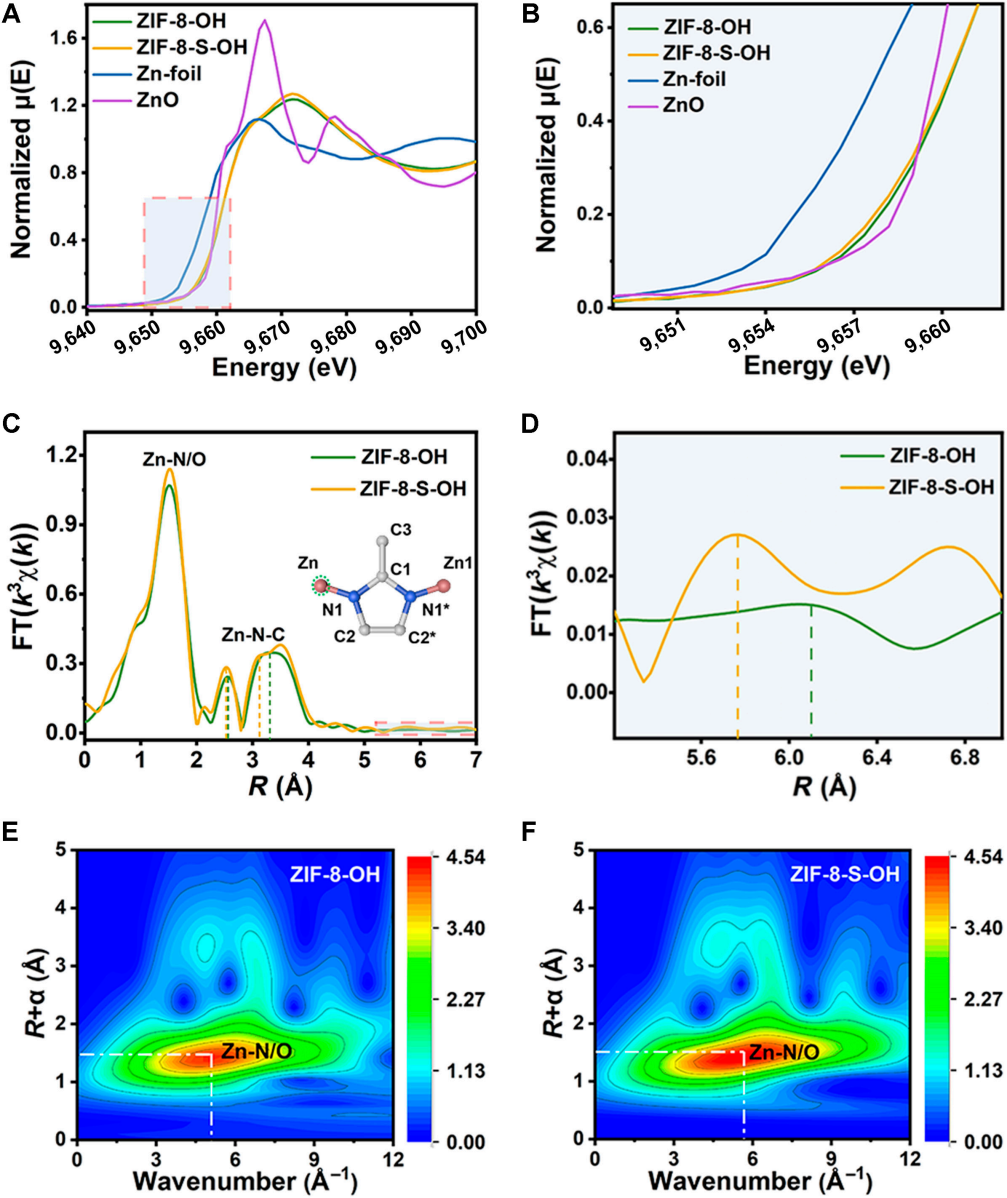
X-ray absorption spectra of samples. (A and B) XANES spectra of Zn K-edge in ZIF-8-OH, ZIF-8-S-OH, Zn-foil, and ZnO. (C) EXAFS of Zn K-edge in ZIF-8-OH and ZIF-8-S-OH. The illustration is a schematic of naming different scattering atoms. (D) EXAFS spectra with high-*R* contributions, and WT of Zn K-edge EXAFS for (E) ZIF-8-OH and (F) ZIF-8-S-OH.

The x-ray absorption spectra were further investigated to reveal the coordination environment of ZIF-8-OH and ZIF-8-S-OH (Fig. 3). In the x-ray absorption near-edge structure (XANES) spectra (Fig. 3A and B), unlike the reported ZIF-8, the pre-edge peaks of ZIF-8-OH and ZIF-8-S-OH were inconspicuous. This observation may be attributed to the 2-methylimidazole (MeIM) twist of the monodentate coordination, which is associated with the grafting of the hydroxyl group onto the framework [42]. The absorption edge of ZIF-8-OH was slightly shifted to the right of ZIF-8-S-OH (Fig. 3B), indicating a higher average valence state of Zn in ZIF-8-OH. Figure 3C and D showed the extended x-ray absorption fine structure (EXAFS) spectra of the 2 samples. The strongest peaks in the R space of ZIF-8-OH and ZIF-8-S-OH were attributed to the first coordination shell of Zn, denoted as Zn-N/O. Although Zn-N and Zn-O cannot be distinguished theoretically by EXAFS, it was observed that the distance of Zn-N/O in ZIF-8-OH was slightly shorter than that in ZIF-8-S-OH, possibly due to the different contributions of shorter Zn-O bonds and longer Zn-N bonds in the 2 samples. The difference in the coordination environment was also evident in the second shell of Zn. The peaks located at 2 to 4 Å were attributed to single and multiple scattering paths related to MeIm [40]. Notably, for ZIF-8-OH and ZIF-8-S-OH, the *R* values of Zn-N-C were different (2.6 Å and 3.3 Å for ZIF-8-OH, 2.5 Å and 3.1 Å for ZIF-8-S-OH). This discrepancy may be attributed to the competitive coordination of the hydroxyl group, causing a portion of the imidazole to become a monodentate ligand, leading to twist along the N1–N1* axis (illustration of Fig. 3C) and increase the path of Zn-N-C. At the same time, the peak at 5.8 Å in ZIF-8-S-OH could be assigned to Zn-Zn, while this peak in ZIF-8-OH shifted to a higher distance (6.1 Å), implying more hydroxyl groups and greater lattice expansion in ZIF-8-OH. To gain further insight into the EXAFS signal, wavelet transform (WT) analysis of ZIF-8-OH and ZIF-8-S-OH was performed, providing more detailed and accurate local structural information. The WT results were visualized as contour plots that combine 2 separate data, including the wave vector (*k*) and the interatomic distance (*R*). Although it was difficult to distinguish Zn-N and Zn-O bonds with similar *R* values from EXAFS, the backscattering atoms could exhibit different oscillations in the *k*-space in the wavelet transform spectra [43,44]. As shown in Fig. 3E and F, the red area showed the contribution of the Zn-N/O coordination shell in ZIF-8-OH and ZIF-8-S-OH. The Zn-N/O of the 2 samples exhibited maximum intensity at approximately 1.5 Å in *R*-space. However, due to the different backscattering atoms (Zn-O-H and Zn-N-C), ZIF-8-OH and ZIF-8-S-OH presented their highest intensity of wavenumber located at 5.1 Å^−1^, and 5.7 Å^−1^, respectively. Considering that the shorter Zn-O bond might have a stronger oscillation at a lower wavenumber [44], it could be speculated that ZIF-8-OH possessed more Zn-O bonds than ZIF-8-S-OH.

### Mechanisms of fabricating CA mimics via trans-functionalization

**Fig. 4.**
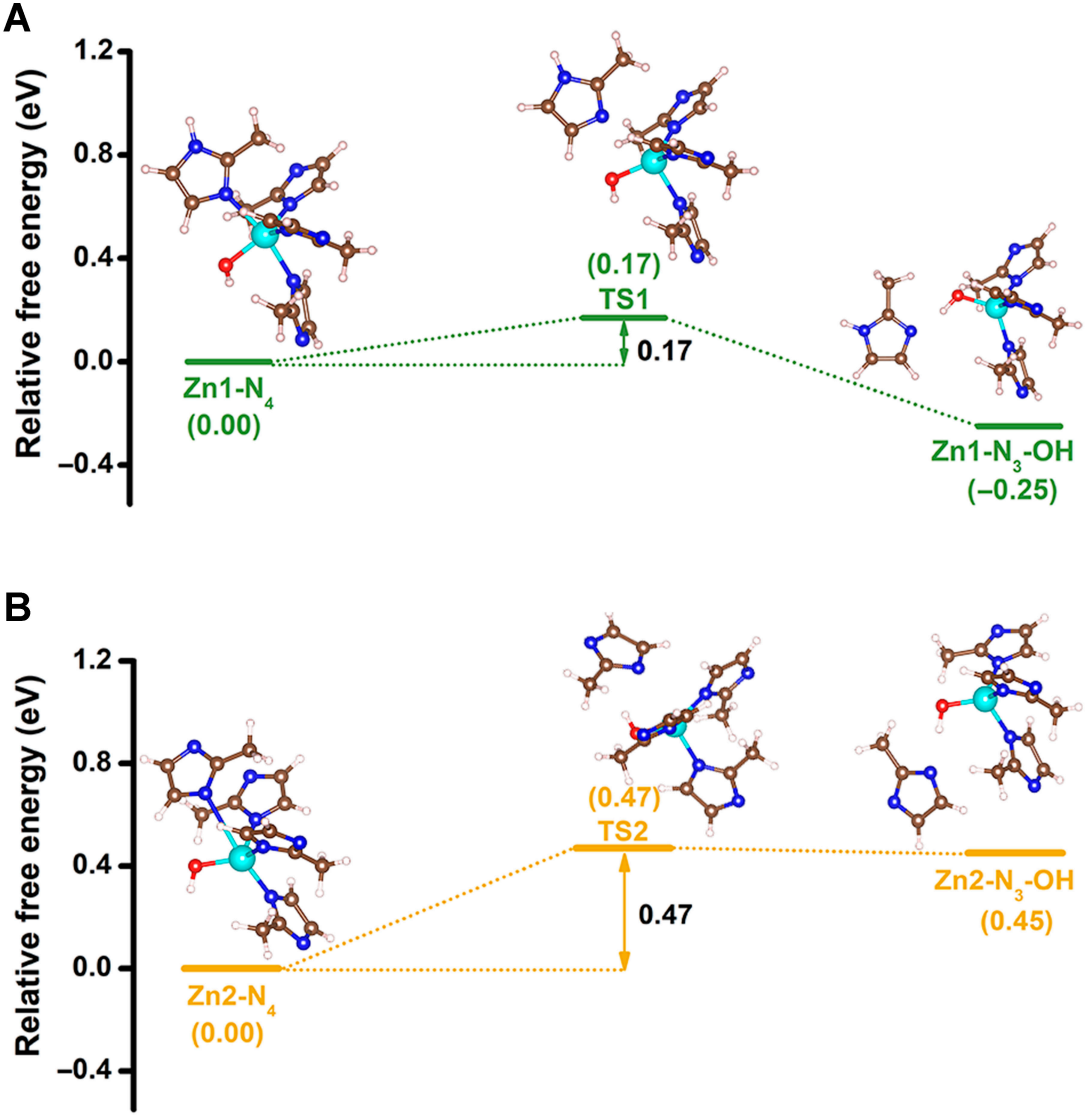
Theoretical calculations. The underlying mechanism of ZIF-8-OH is constructed by the structural transformation of ZIF-L. Free energy profiles along the pathway for the synthesis of (A) Zn1-N_3_-OH and (B) Zn2-N_3_-OH. The illustrations show the optimized structures of the initial reactants, transition states, and final products involved in the synthesis path (C, gray; H, white; O, red; N, blue; and Zn, cyan).

DFT calculations were employed to reveal the underlying mechanism of CA mimics constructed by the structural transformation of ZIF-L. The periodic structural unit of ZIF-8 with sodalite (SOD) topology is fabricated by the coordination of one Zn^2+^ with 4 N atoms of MeIm [45]. ZIF-L is a 2-dimensional layered material with the same building blocks as ZIF-8 but with a distinct feature: part of the MeIm molecule in ZIF-L remains protonated, resulting in the formation of hydrogen bonds that connect the layers of the 2-dimensional ZIF-L [46]. Thus, ZIF-L contains 2 types of Zn clusters: one coordinated with 3 deprotonated imidazoles and one protonated imidazole (denoted as Zn1-N_4_), and the other coordinated with 4 deprotonated imidazoles (denoted as Zn2-N_4_). Two reaction pathways involving all the transition states (TS) are shown in Fig. 4. When Zn1-N_4_ is attacked by OH^−^, a lower energy barrier of 0.17 eV is required to reach the transition state TS1 compared to Zn2-N_4_ (0.47 eV). This difference in energy barriers can be attributed to the asymmetric molecular structure of Zn1-N_4_. The π−d conjugated coordination between protonated imidazole and Zn^2+^ was significantly weakened by the hydrogen bond between protonated imidazole and OH^−^ [46], making it more favorable for the subsequent bond breaking reaction to occur at the site of protonated imidazoles. After that, the unsaturated Zn that coordinates with the hydroxyl group would be spontaneous, resulting in a negative energy barrier throughout the entire structural transformation process from Zn1-N_4_ to Zn1-N_3_-OH. Correspondingly, energy barriers of 0.45 eV are required for the transformation from Zn2-N_4_ to Zn2-N_3_-OH. These findings suggest that hydroxyl group coordination with the interlayer Zn cluster forms the CA-like active site of Zn-N_3_-OH. Besides, since the Zn cluster in ZIF-8 is consistent with Zn2-N_4_, the calculations indicate that modifying hydroxyl groups through structural transformation has a lower energy barrier than modifying hydroxyl groups by post-functionalization, which was in good agreement with the experimental results.

### Biomimetic catalytic performance

**Fig. 5.**
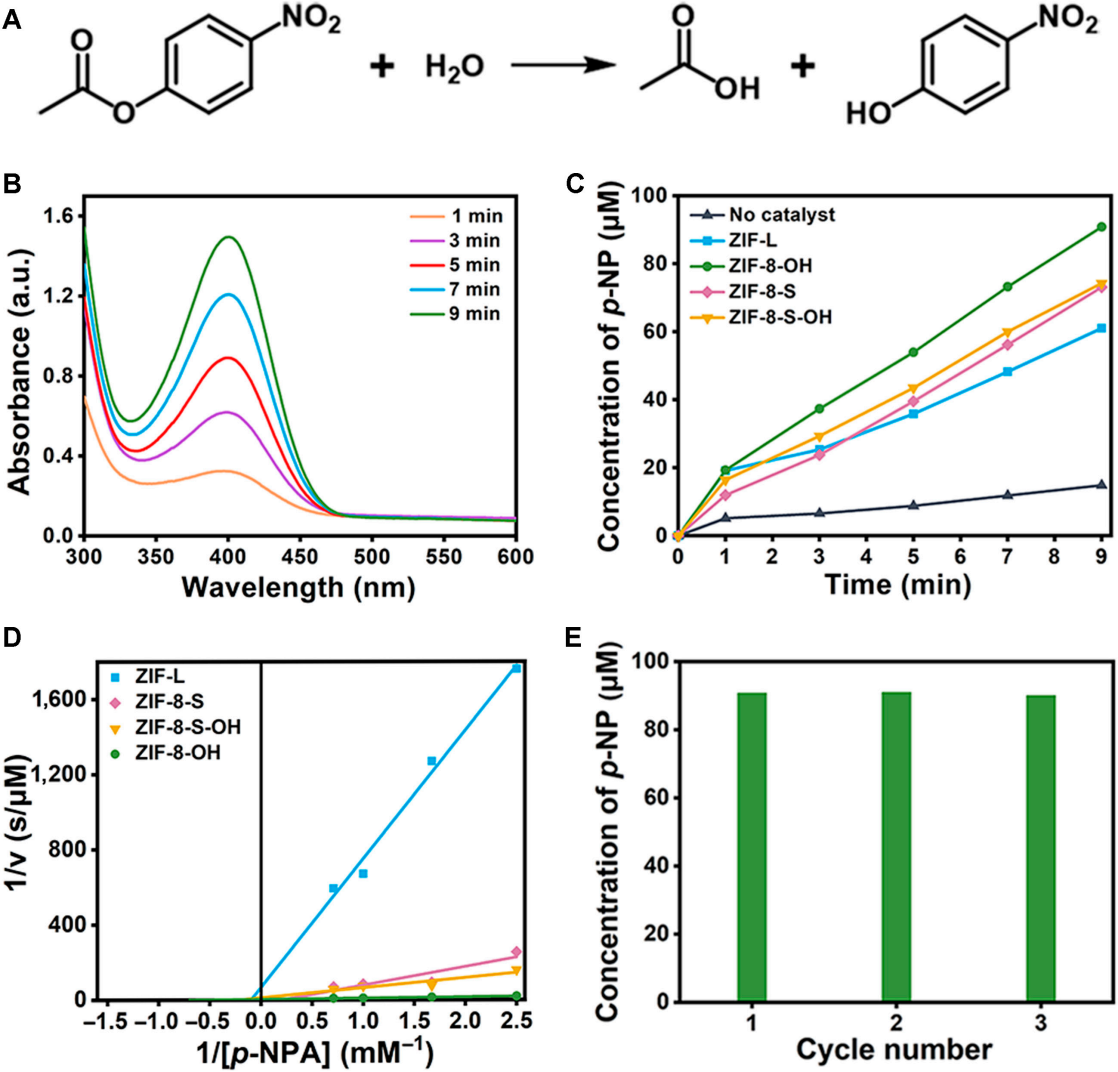
Evaluation of biomimetic catalytic activity. (A) Scheme of the *p*-NPA hydrolysis reaction. (B) UV–Vis spectra of reaction solution within 9 min in the presence of ZIF-8-OH. (C) Kinetic curves of the hydrolysis catalysis of *p*-NPA using no catalyst and a series of carbonic anhydrase mimics as catalysts. (D) Double reciprocal plots of *p*-NPA concentrations vs. activities for a series of carbonic anhydrase mimics and (E) reusability of ZIF-8-OH.

The biomimetic activity of CA mimics was evaluated through the catalytic performance of *p*-NPA hydrolysis (Fig. 5A). To determine the concentration of *para*-nitrophenol (*p*-NP) which is the hydrolysis product of *p*-NPA, a calibration curve was plotted using ultraviolet–visible (UV–Vis) spectroscopy at 400 nm for a series of *p*-NP solutions at set concentrations (Fig. S7). The self-decomposition of *p*-NPA in the absence of the catalyst was also tested, alongside the biomimetic activities of ZIF-L, ZIF-8-L, ZIF-8-S, and ZIF-8-S-OH (Fig. 5B and C and Figs. S8 to S12). In the absence of the mimic CA catalyst, the concentration of the *p*-NP hardly increased with the prolongation of the reaction time, suggesting spontaneous hydrolysis of *p*-NPA with relatively low efficiency. ZIF-8-OH exhibited the highest catalytic performance among the biomimetic catalysts, attributed to the synergistic effect of more active sites and obvious mesopores present in it. Specifically, mesopores were generated along with the fabrication of active sites. The high density of active sites directly facilitated the hydrolysis of *p*-NPA, whereas the mesopores enhances the accessibility of reactants to the active sites and reduces mass transfer resistance during the reaction process. ZIF-8-S-OH showed a trivial increase in activity compared to ZIF-8-S, reflecting the few active centers generated by post-functionalization. In contrast, ZIF-8-L, lacking the grafted hydroxyl group, demonstrated significantly lower catalytic efficiency than ZIF-8-OH. Notably, ZIF-L and ZIF-8-S also exhibited some catalytic activity, which can be attributed to the presence of defects on their surfaces. These defects allow H_2_O to adsorb and generate a small amount of Zn-N_3_-OH active units. Additionally, the surface defects of ZIF-L and ZIF-8-S can provide Lewis acid sites like Zn^2+^, Brønsted acid sites (-NH groups) from MeIm, and base sites (N-extremities), which can also enhance the hydrolysis reaction [47,48]. The control experiments of Zn^2+^ and MeIm were conducted respectively at the same concentration as ZIF-8-OH. The detected catalytic activities were higher than the spontaneous hydrolysis activity of *p*-NPA but significantly lower than that of the 4 ZIFs studied, especially ZIF-8-OH (Figs. S13 to S15). It indicated that the hydroxyl structure should be the pivotal factor determining the high catalytic activity of ZIFs. The catalytic activity of ZIF-L shown in Fig. 5C was lower than that of ZIF-8-S, while the catalytic activity of ZIF-8-OH derived from ZIF-L was significantly greater than that of ZIF-8-S-OH derived from ZIF-8-S. This supported the idea that trans-functionalization produced more active sites of Zn-N_3_-OH in ZIFs than post-functionalization, thereby enhancing the catalytic reaction of *p*-NPA hydrolysis.

The enzymatic Michaelis–Menten curves for ZIF-8-OH, ZIF-8-S-OH, ZIF-8-S, and ZIF-L were measured by varying the substrate concentration while maintaining a constant catalyst concentration (Fig. 5D). The *K*_m_ values were as follows: ZIF-L at 10.2 mM, ZIF-8-S at 4.93 mM, ZIF-8-OH at 1.47 mM, and ZIF-8-S-OH at 4.42 mM. The lower *K*_m_ values for ZIF-8-OH and ZIF-8-S-OH, compared to ZIF-8-S and ZIF-L, indicated that the hydroxyl modification enhanced the affinity of the catalyst to the substrate. Additionally, the decrease in *K*_m_ from ZIF-L to ZIF-8-OH was much greater than that from ZIF-8-S to ZIF-8-S-OH. These results suggested that the process of trans-functionalization grafted more hydroxyl groups onto ZIFs than that of post-functionalization, which significantly increased the affinity of substrates. Correspondingly, the *V*_max_ values of the 4 ZIFs were as follows: ZIF-8-OH (0.24 μM/s), ZIF-8-S-OH (0.08 μM/s), ZIF-8-S (0.05 μM/s), and ZIF-L (0.015 μM/s), in order from highest to lowest. These values align with the CA-like catalytic activity shown in Fig. 5C, further demonstrating that ZIF-8-OH, with more active centers, had superior CA catalytic performance compared to ZIF-8-S-OH. Moreover, the reusability of ZIF-8-OH was also evaluated as shown in Fig. 5E. The negligible decrease in catalytic performance over 3 cycles and the structural integrity after the reaction (Fig. S16) confirmed the good stability of ZIF-8-OH.

## Discussion

In summary, we have proposed a novel strategy of trans-functionalization to fabricate CA-like catalysts through the structural transformation from ZIF-L to ZIF-8. During this progress, OH^−^ not only deprotonated the ZIF-L to trigger the structural transformation but also competed with imidazoline N to coordinate with Zn, generating CA-mimicking active centers of Zn-N_3_-OH. Compared to ZIF-8-S-OH fabricated by traditional post-functionalization, the as-prepared ZIF-8-OH exhibited a lower reaction energy barrier and more accessible active centers, leading to higher catalytic efficiency in *p*-NPA hydrolysis. More importantly, this strategy harnesses the structural advantages of MOFs and offers a promising perspective for the development of highly efficient enzyme mimics within MOFs.

## Materials and Methods

### Materials and chemicals

All the reagents were used as received without further purification. Zinc nitrate hexahydrate (Zn(NO_3_)_2_·6H_2_O, 99%, Sigma-Aldrich), 2-methylimidazole (C_4_H_6_N_2_, 98%, Sigma-Aldrich), TBAOH (40wt% in H_2_O, Macklin), *p*-NPA (98%, Macklin), phosphate-buffered saline (PBS) buffer solution (10 mM, pH 7.2, ACMEC), methanol (CH_3_OH, 99.5%, Sinopharm), ethanol (C_2_H_5_OH, 99.5%, Sinopharm), and DMF (99%, Sinopharm) were used in the study.

### Synthesis of ZIF-L

According to the previously reported literature [37], ZIF-L was first synthesized. Forty milliliters of Zn(NO_3_)_2_·6H_2_O (0.59 g, 50 mM) aqueous solution was added to the 40-ml 2-methylimidazole (1.3 g, 396 mM) aqueous solution under stirring, and the mixture was continuously stirred at room temperature for 4 h. The product was isolated by centrifugation (10,000 rpm, 10 min), washed twice with ethanol and once with methanol, and dried by vacuum evaporation at 70 °C overnight.

### Synthesis of ZIF-8-OH

An aqueous solution of TBAOH (1.24 ml, 1.9 mmol) was added dropwise to ZIF-L (100 mg, 0.37 mmol) under a nitrogen atmosphere, and the mixture was stirred for 12 h. Then, the solid was collected by centrifugation (8,000 rpm, 5 min) and washed several times with water to remove excess TBA^+^ and OH^−^. The sample was dried by vacuum evaporation at 70 °C overnight.

### Synthesis of ZIF-8-L

ZIF-L (25 mg) was added to a mixed solution of DMF (24 ml) and ethanol (8 ml). Subsequently, the solution was continuously ultrasonicated for 5 min. The reaction was heated to 70 °C and then kept for 24 h. The product was collected by centrifuging at 8,000 rpm for 10 min and washed several times with ethanol and then vacuum-dried overnight.

### Synthesis of ZIF-8-S

A 50-ml methanol solution of Zn(NO_3_)_2_·6H_2_O (25 mM) and a 50-ml methanol solution of 2-methylimidazole (25 mM) were mixed briefly in a glass vial to form a homogeneous solution. The reaction mixture was kept at room temperature for 24 h without stirring. Subsequently, the precipitates were achieved by centrifugation (8,000 rpm, 5 min), washed several times with methanol, and then vacuum-dried overnight to obtain ZIF-8-S.

### Synthesis of ZIF-8-S-OH

Under a nitrogen atmosphere, an aqueous solution of TBAOH (1.24 ml, 1.9 mmol) was added dropwise to ZIF-8 (84 mg, 0.37 mmol), and the mixture was kept at room temperature and stirred for 12 h. Subsequently, the product was achieved by centrifugation (8,000 rpm, 5 min) and washed several times with water to remove excess TBA^+^ and OH^−^. The product was dried by vacuum evaporation at 70 °C overnight.

### Hydrolysis of *p*-NPA

The catalytic performance of all catalysts (ZIF-L, ZIF-8-OH, ZIF-8-L, ZIF-8-S, and ZIF-8-S-OH) was evaluated by monitoring the hydrolysis of *p*-NPA at 400 nm by UV–Vis spectroscopy. In a typical experiment, 17 mg of *p*-NPA and 9.6 mg of catalysts were added sequentially into a 100-ml glass vial containing 96 ml of 10 mM PBS buffer (pH 7.2). The mixture was sonicated to afford a homogeneous suspension. The reaction was allowed to stir at 25 °C. After the reaction, the reaction product was analyzed by the absorbance at 400 nm in the UV–Vis spectrum. Meanwhile, the above experimental operations were carried out without the catalyst to test the catalytic hydrolysis performance of *p*-NPA.

### Evaluation of kinetic parameters

The catalytic hydrolysis activity of *p*-NPA was tested by ZIF-L, ZIF-8-S, ZIF-8-OH and ZIF-8-S-OH at different concentrations of the substrate that ranged from 0.4 to 1.4 mM, respectively. The values of Michaelis–Menten constant (*K*_m_) and the maximum rate of enzymatic reaction (*V*_max_) were calculated based on the relationship between the substrate concentration and the hydrolysis reaction rate.

### Recycling experiments of hydrolysis of *p*-NPA reaction

The mixture obtained after the hydrolysis of *p*-NPA catalyzed by ZIF-8-OH was centrifuged and washed with water. Then, the catalyst was dried at room temperature for the next catalytic cycle. The catalytic cycle is carried out under the same conditions as *p*-NPA hydrolysis.

### Characterization

The morphology of the samples was studied using a field emission scanning electron microscope (JEOL JSM-7600). The phase of obtained samples was recorded by an x-ray powder diffractometer (Bruker D8 Advance). Nitrogen adsorption isotherms of the samples were measured by the MicroActive for ASAP 2020-4 built-in software at 77 K up to 1 bar. Before measurements, all samples were activated under vacuum at 393 K for 12 h. The Brunauer–Emmett–Teller surface area, pore volume, and pore size were obtained by analyzing nitrogen adsorption and desorption isotherms with the MicroActive for ASAP 2020-4 built-in software. The nitrogen content of the obtained samples was characterized by a commercial CHNS analyzer. The zinc content of the samples was analyzed by an inductively coupled plasma mass spectrometer (Agilent S370). The Zn-OH bond was measured by diffuse reflectance infrared Fourier transform spectroscopy (Digilab FTS-60 equipped with a KBr beamsplitter and a mercury cadmium telluride detector). The production analysis was performed using UV–Vis spectrophotometry (UV-1750) spectra.

### Computational details

DFT calculations of the molecular structures in vacuum were performed using the Vienna ab-initio Simulation Package [49]. The chemical structures were optimized by the Perdew–Burke–Ernzerhof exchange-correlation functional [50] and the projector-augmented wave method [51]. The wave functions were expanded using a plane-wave basis set with a cutoff of 400 eV. The molecular systems were situated in vacuum in a cubic unit cell (lattice parameter: *a* = 20 Å). Due to the large simulation cell, only the Γ point was applied to sample the Brillouin zone. All the atoms in the chemical structures were optimized until the ion force was less than 0.01 eV/Å. The van der Waals correction by Grimme [52] was used to consider the weak interactions. The activation energy was computed by the nudged elastic band method [53].

## Data Availability

All data needed to evaluate the conclusions in the paper are present in the paper and the Supplementary Materials. Additional data related to this paper may be requested from the authors.
