# Supplementary figures and images for: A Structural Mimic of Carbonic Anhydrase in Zeolitic Imidazolate Frameworks via Trans-functionalization for Enhancing Hydrolytic Activity
Source: Research (Wash D C). 2024 Aug 9;7:0434. doi: 10.34133/research.0434 (PMC11310446; doi:10.34133/research.0434)

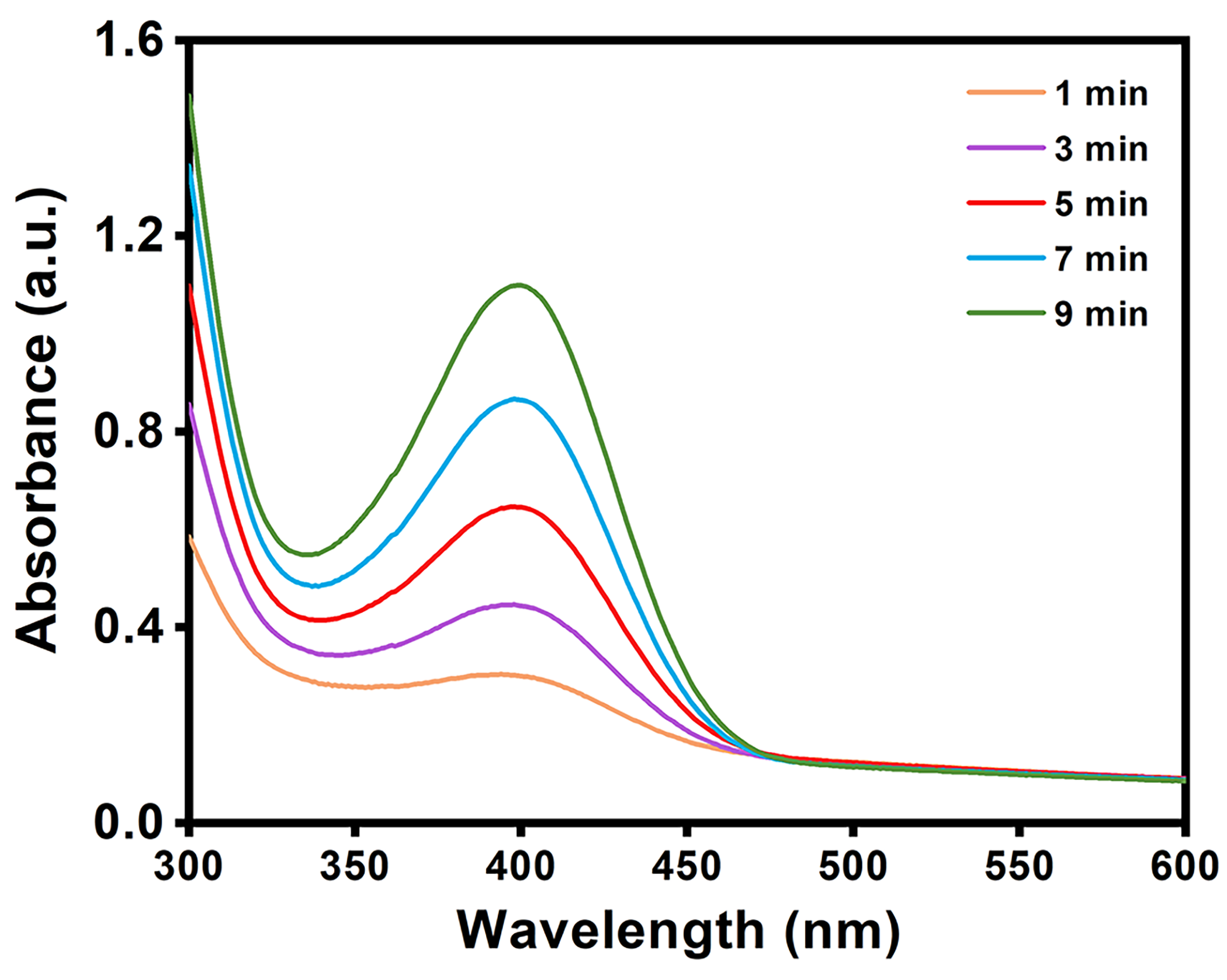

Supplement: Supplementary 1 — Figs. S1 to S16 [file research.0434.f1.zip › Fig. S10.tif]

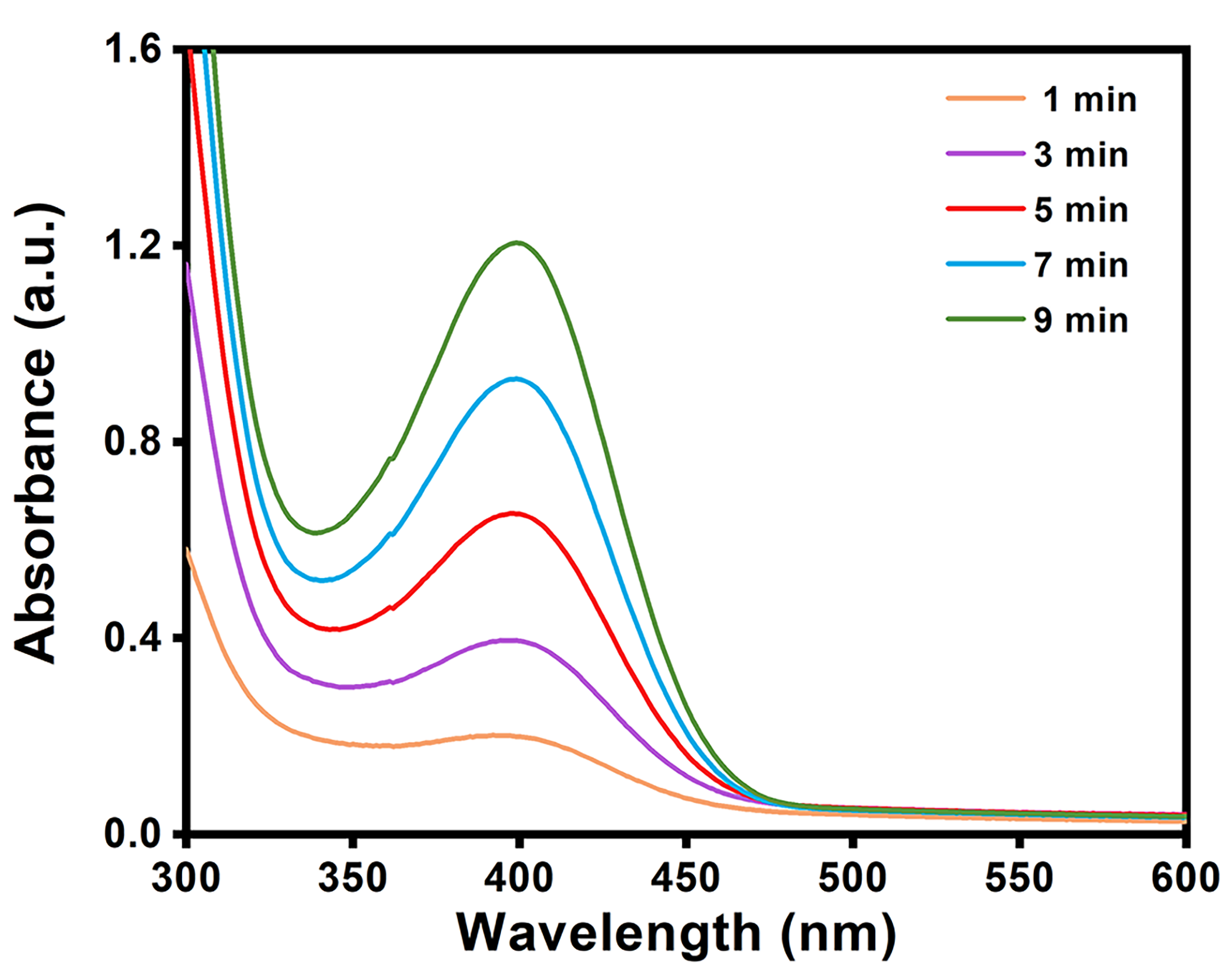

Supplement: Supplementary 1 — Figs. S1 to S16 [file research.0434.f1.zip › Fig. S11.tif]

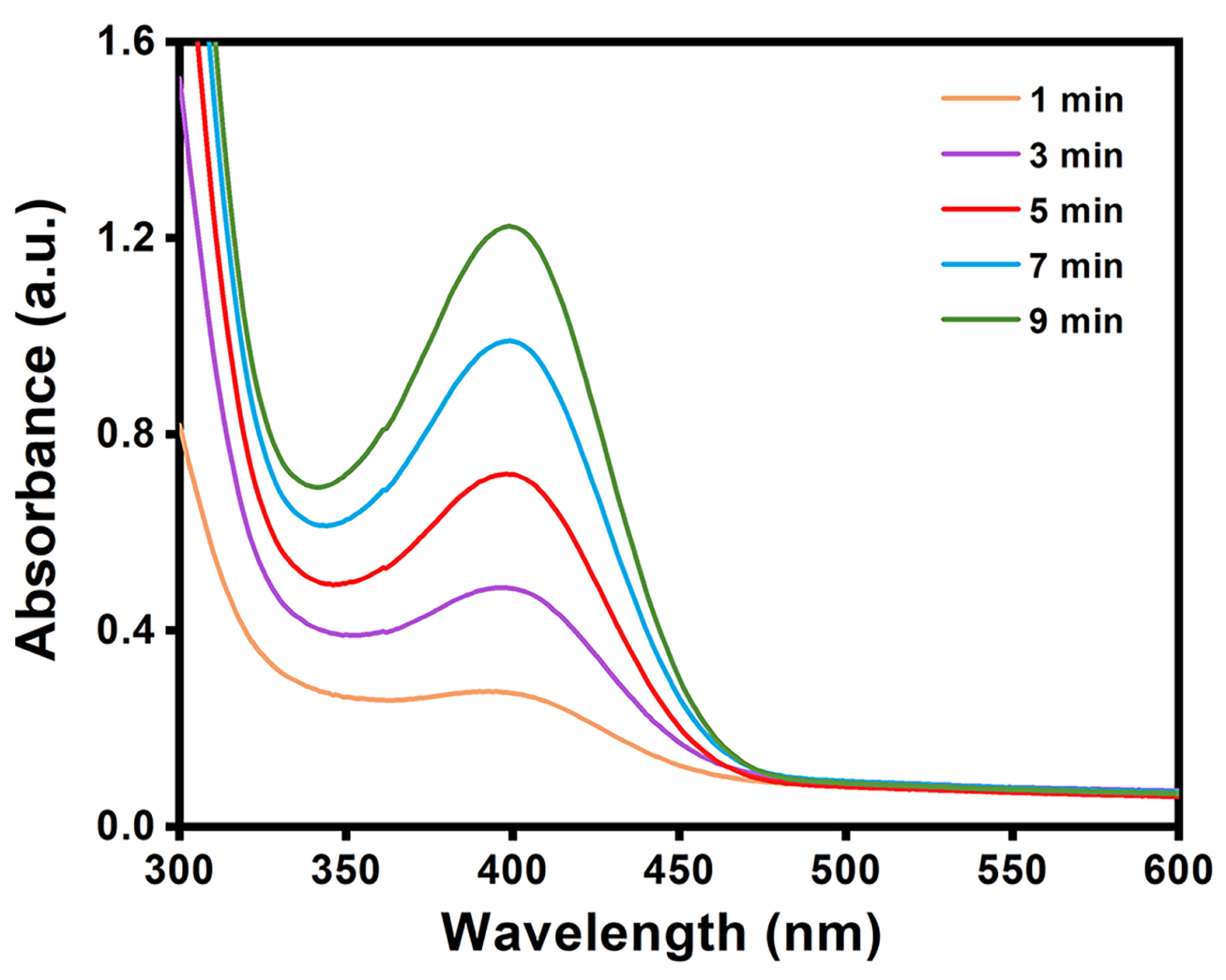

Supplement: Supplementary 1 — Figs. S1 to S16 [file research.0434.f1.zip › Fig. S12.tif]

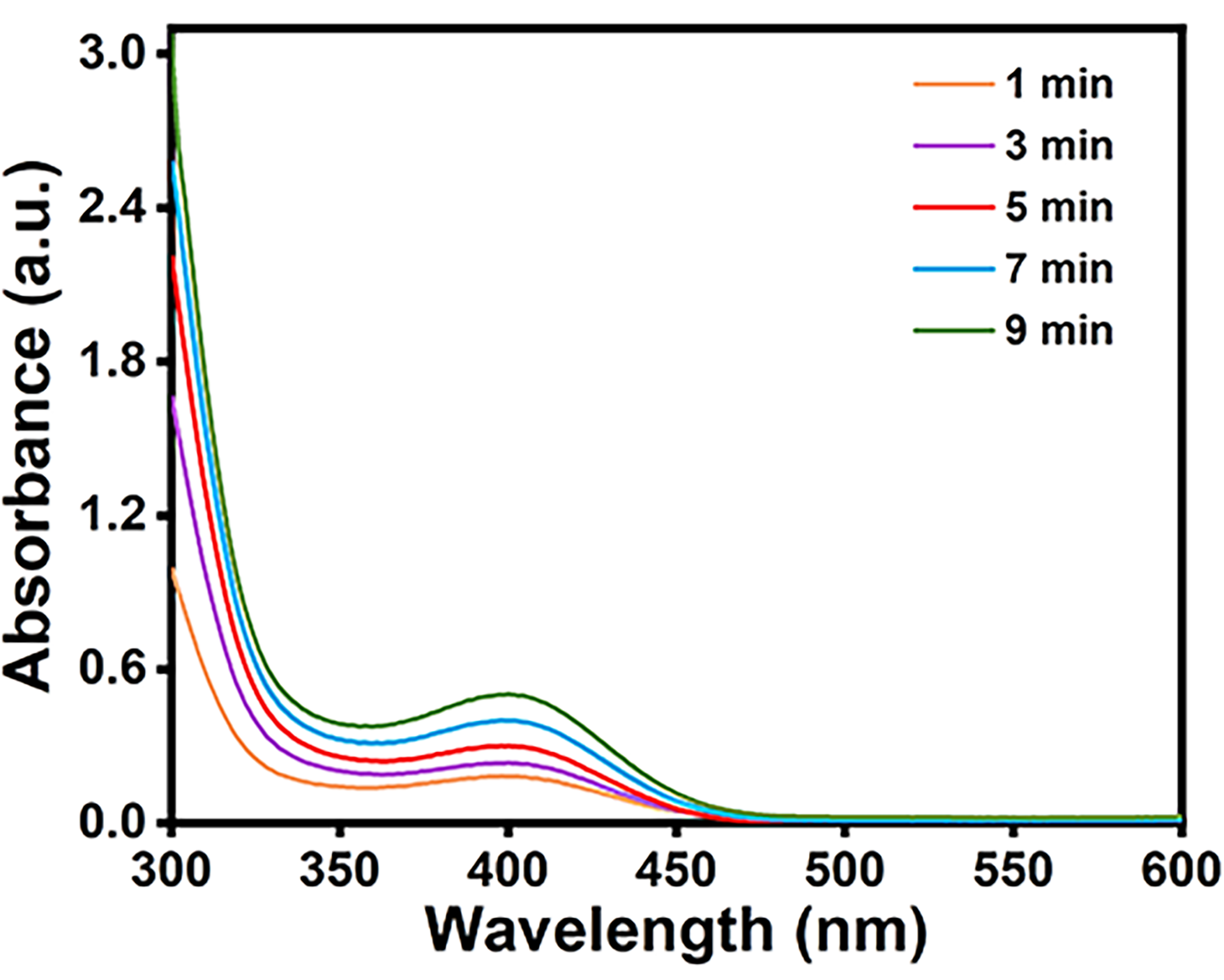

Supplement: Supplementary 1 — Figs. S1 to S16 [file research.0434.f1.zip › Fig. S13.tif]

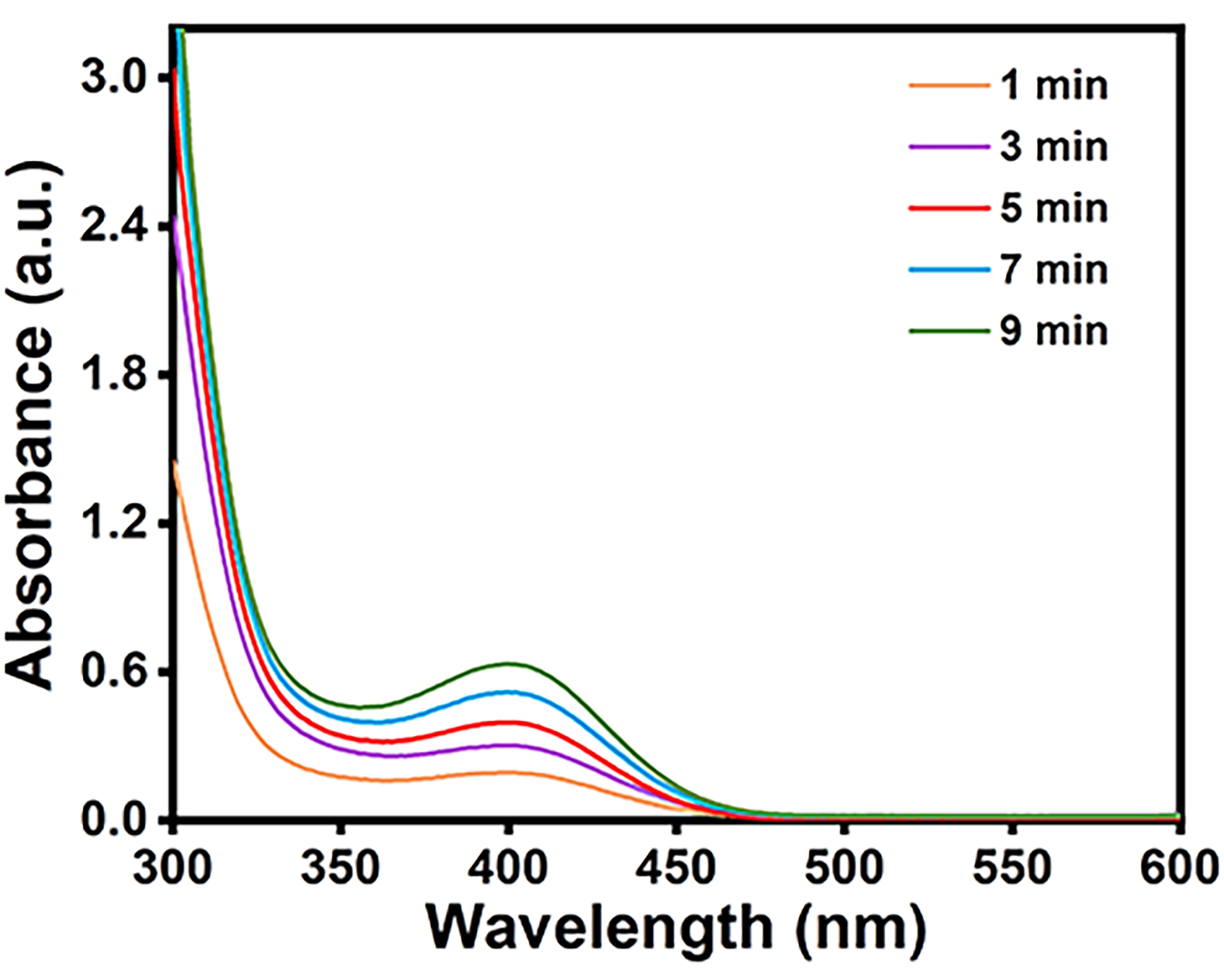

Supplement: Supplementary 1 — Figs. S1 to S16 [file research.0434.f1.zip › Fig. S14.tif]

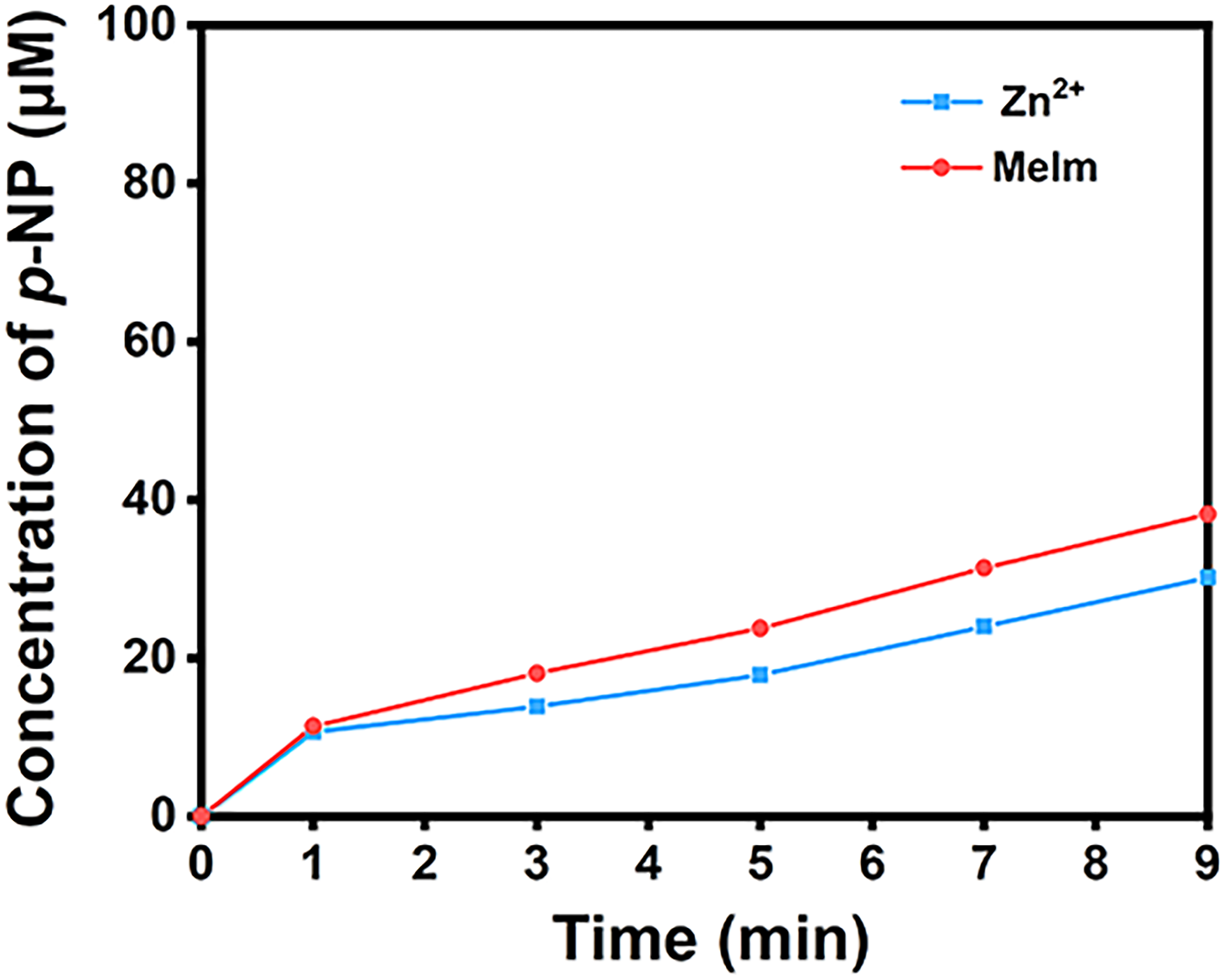

Supplement: Supplementary 1 — Figs. S1 to S16 [file research.0434.f1.zip › Fig. S15.tif]

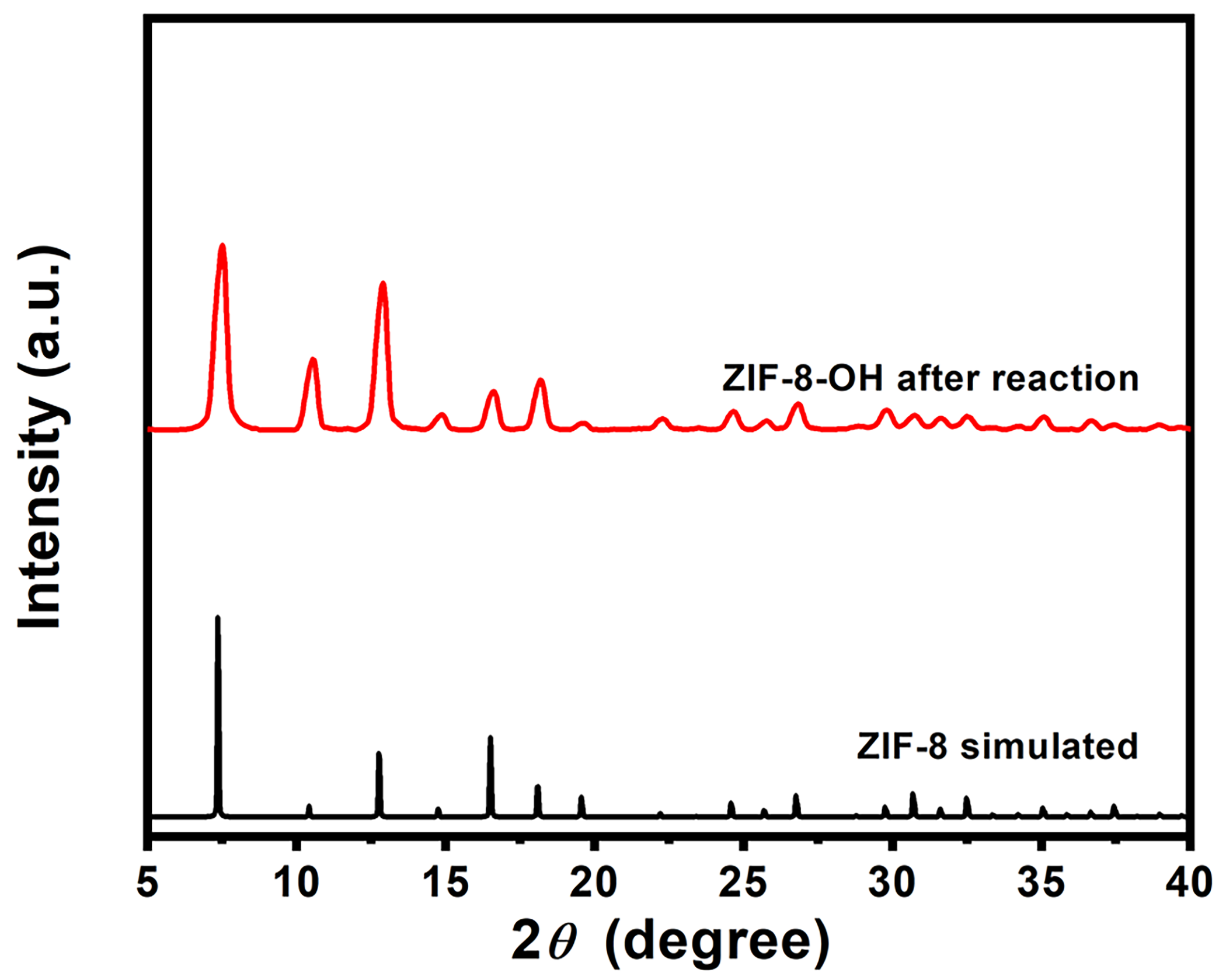

Supplement: Supplementary 1 — Figs. S1 to S16 [file research.0434.f1.zip › Fig. S16.tif]

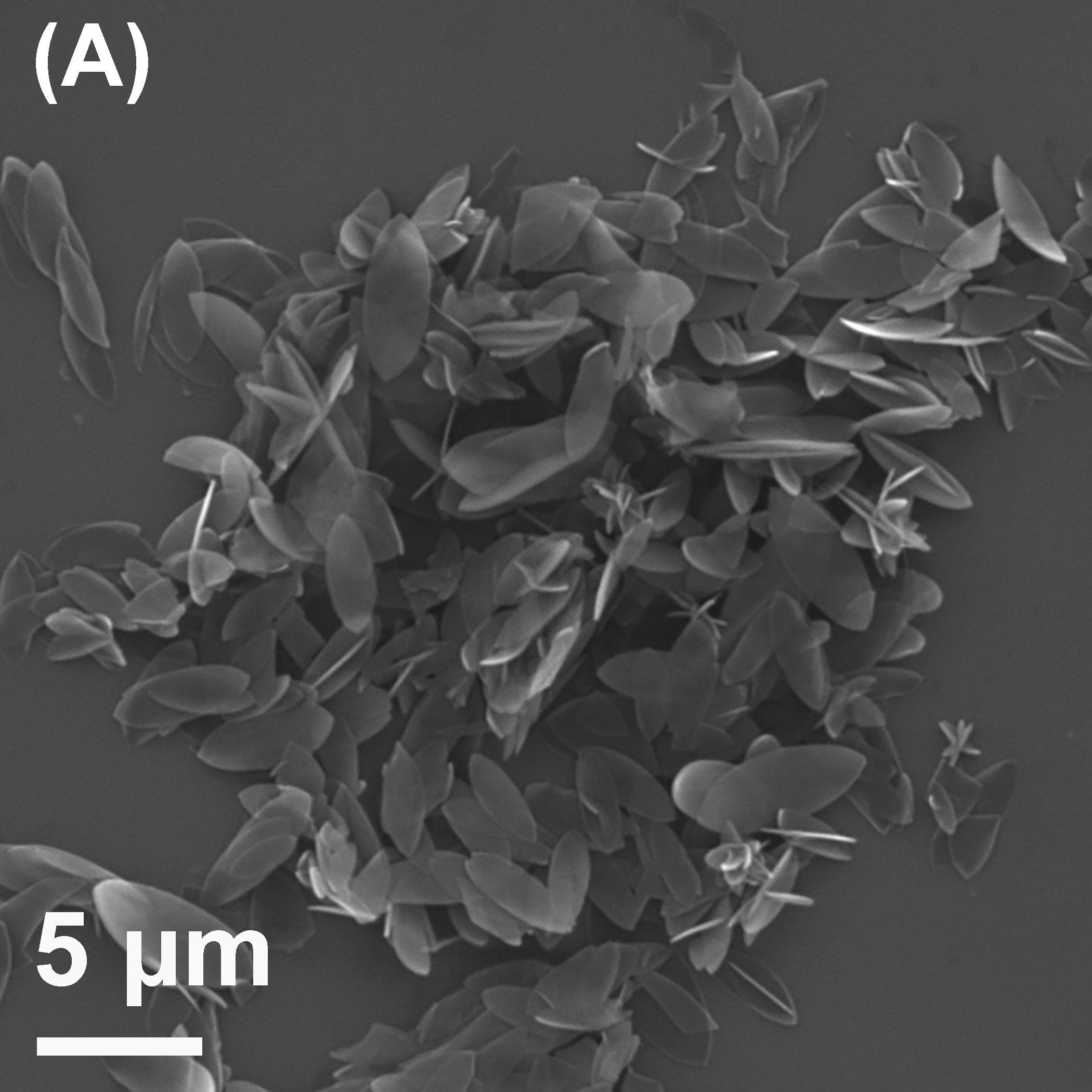

Supplement: Supplementary 1 — Figs. S1 to S16 [file research.0434.f1.zip › Fig. S1A.tif]

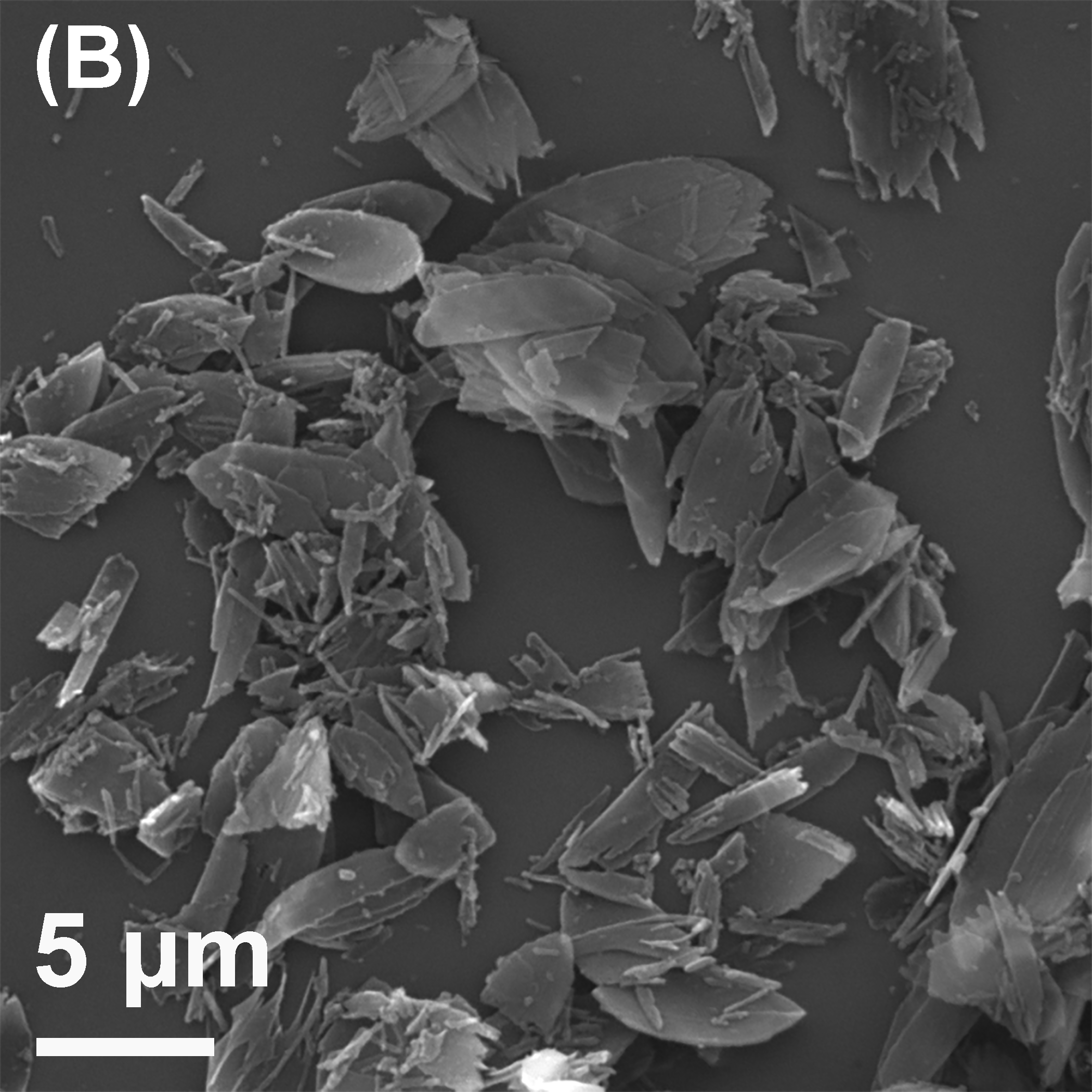

Supplement: Supplementary 1 — Figs. S1 to S16 [file research.0434.f1.zip › Fig. S1B.tif]

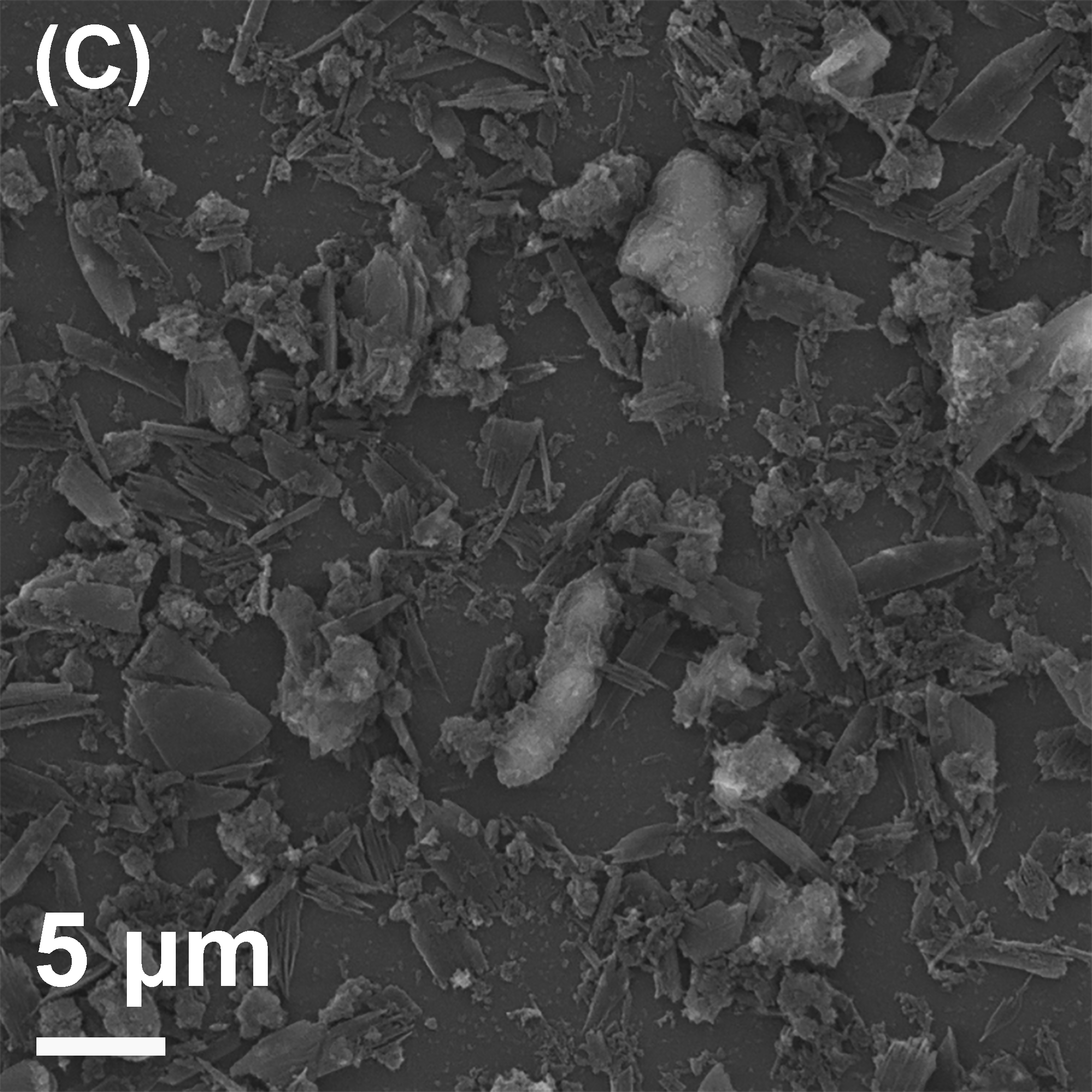

Supplement: Supplementary 1 — Figs. S1 to S16 [file research.0434.f1.zip › Fig. S1C.tif]

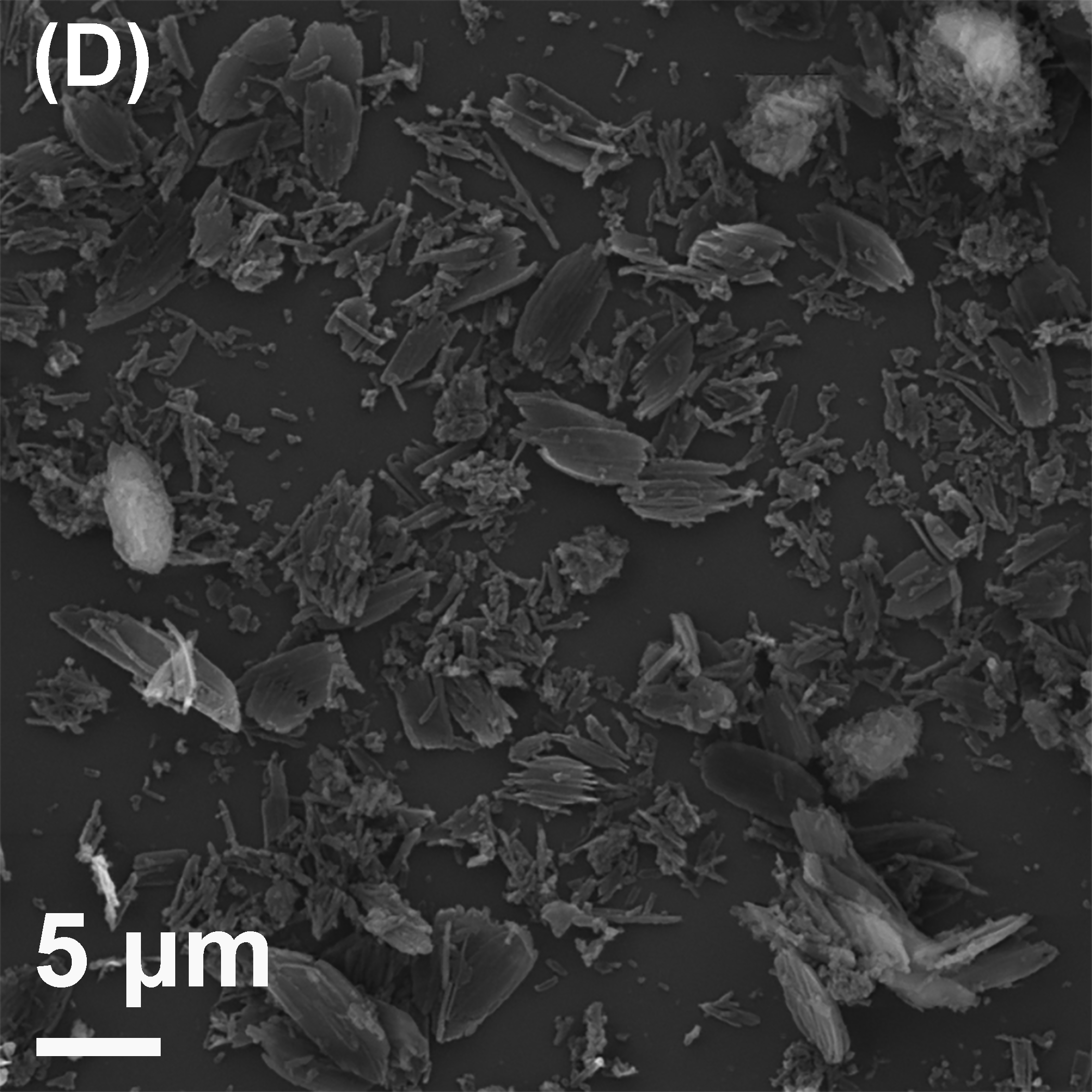

Supplement: Supplementary 1 — Figs. S1 to S16 [file research.0434.f1.zip › Fig. S1D.tif]

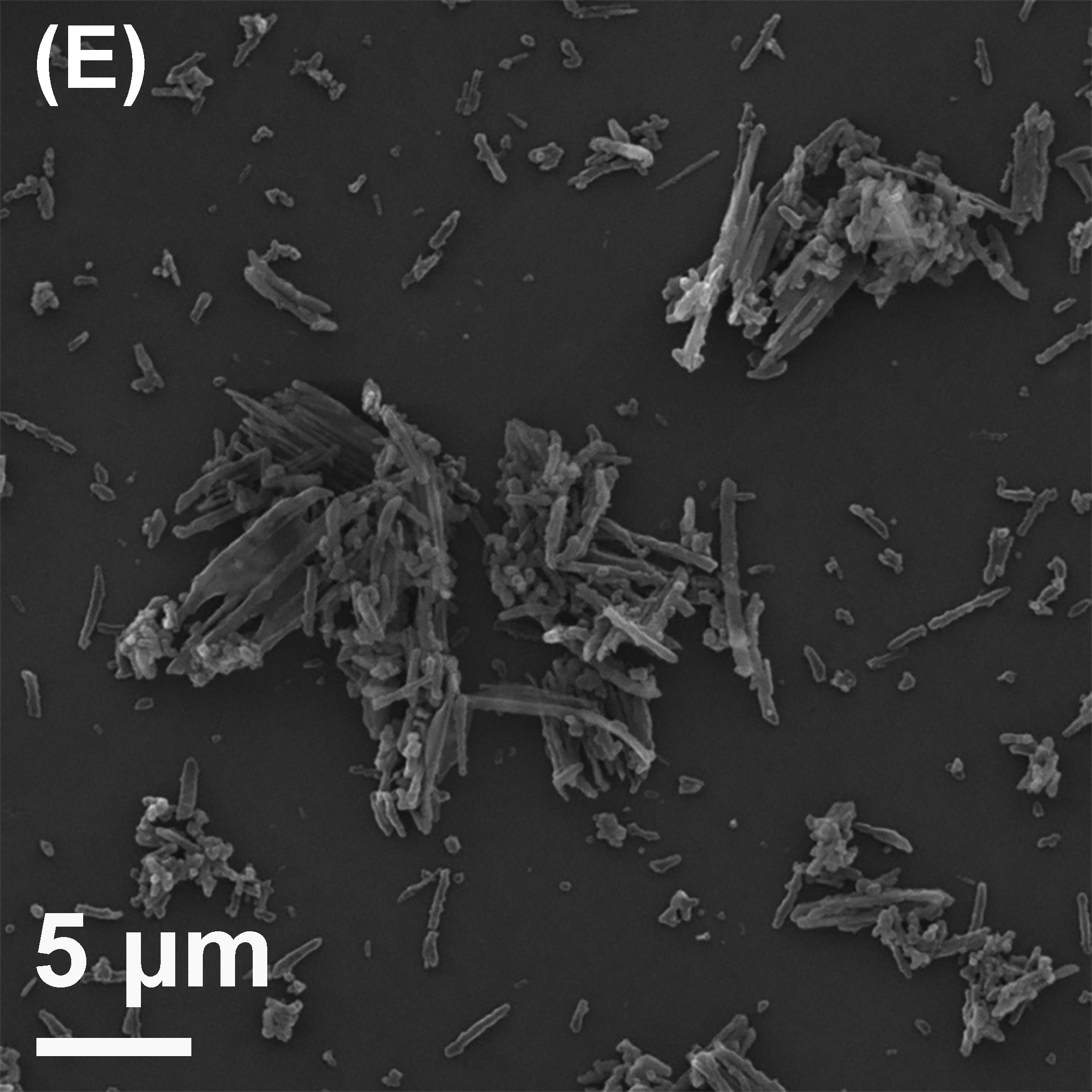

Supplement: Supplementary 1 — Figs. S1 to S16 [file research.0434.f1.zip › Fig. S1E.tif]

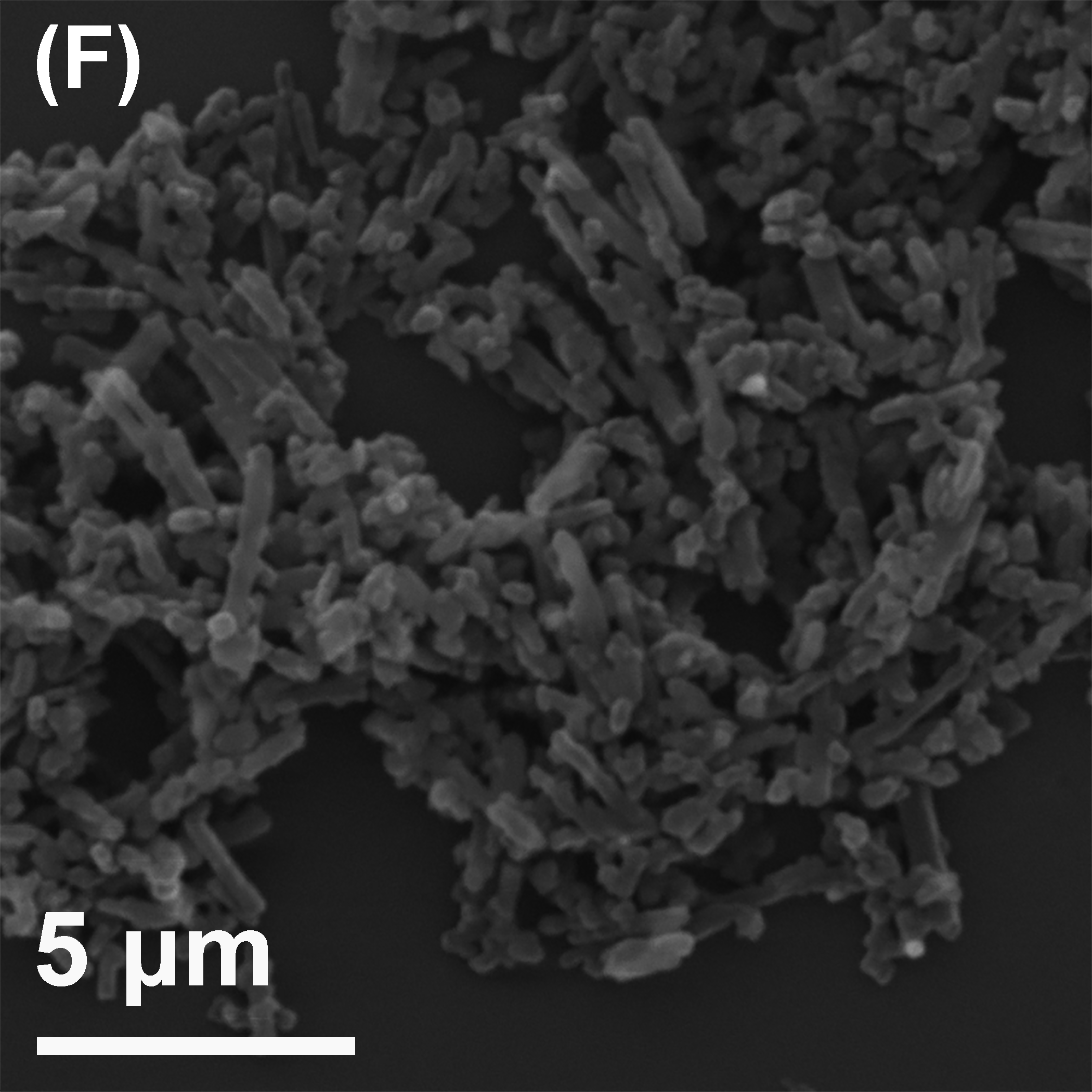

Supplement: Supplementary 1 — Figs. S1 to S16 [file research.0434.f1.zip › Fig. S1F.tif]

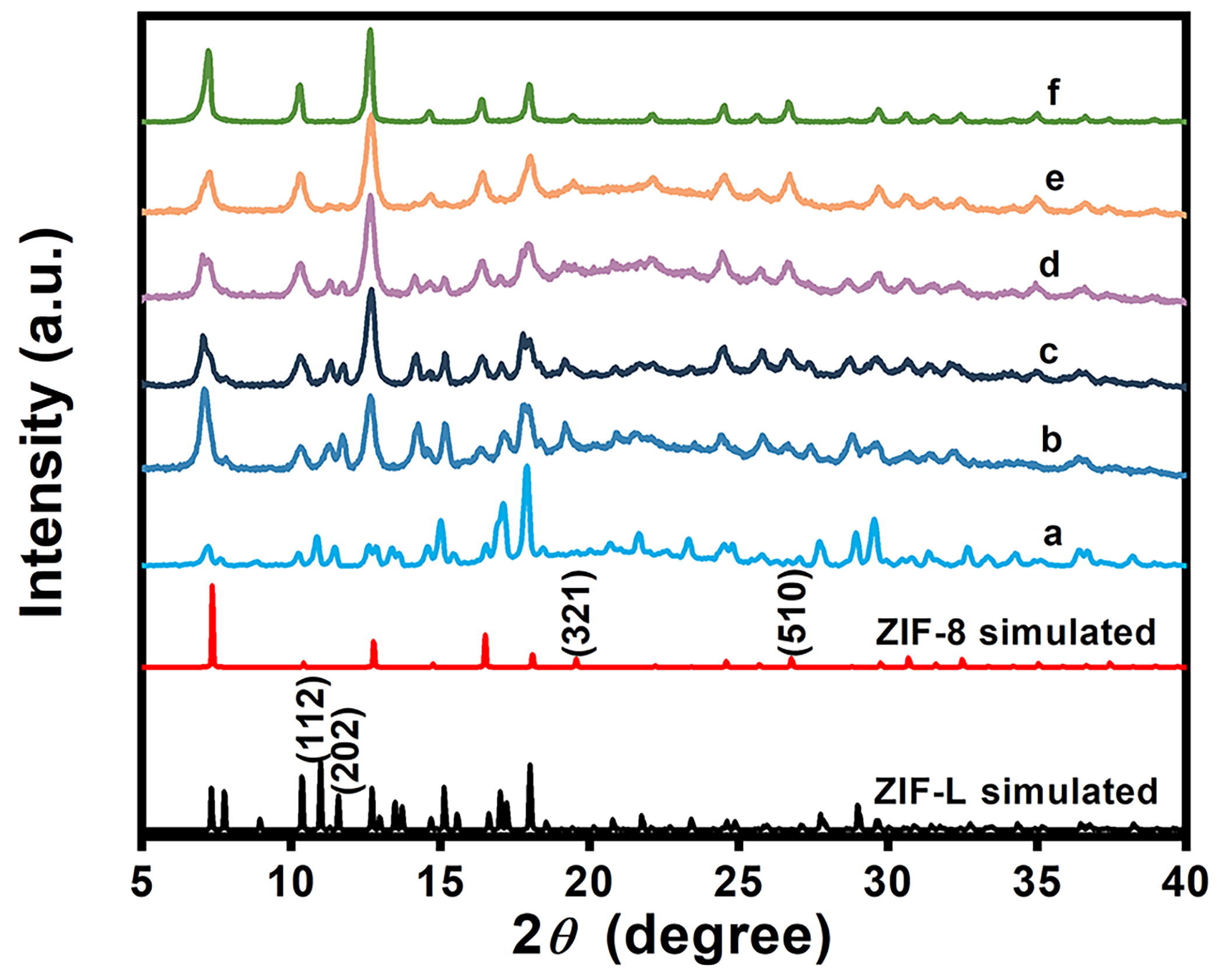

Supplement: Supplementary 1 — Figs. S1 to S16 [file research.0434.f1.zip › Fig. S2.tif]

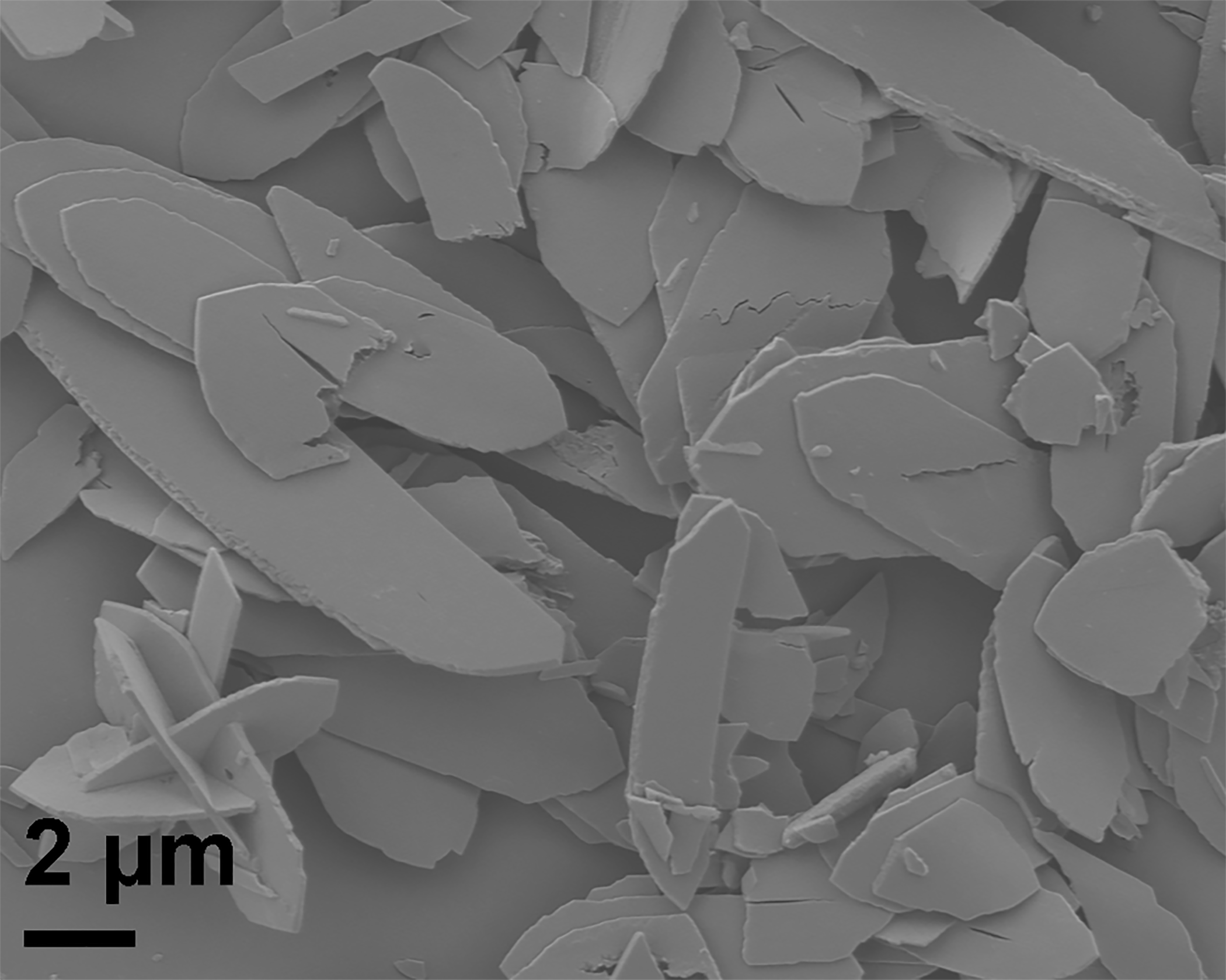

Supplement: Supplementary 1 — Figs. S1 to S16 [file research.0434.f1.zip › Fig. S3.tif]

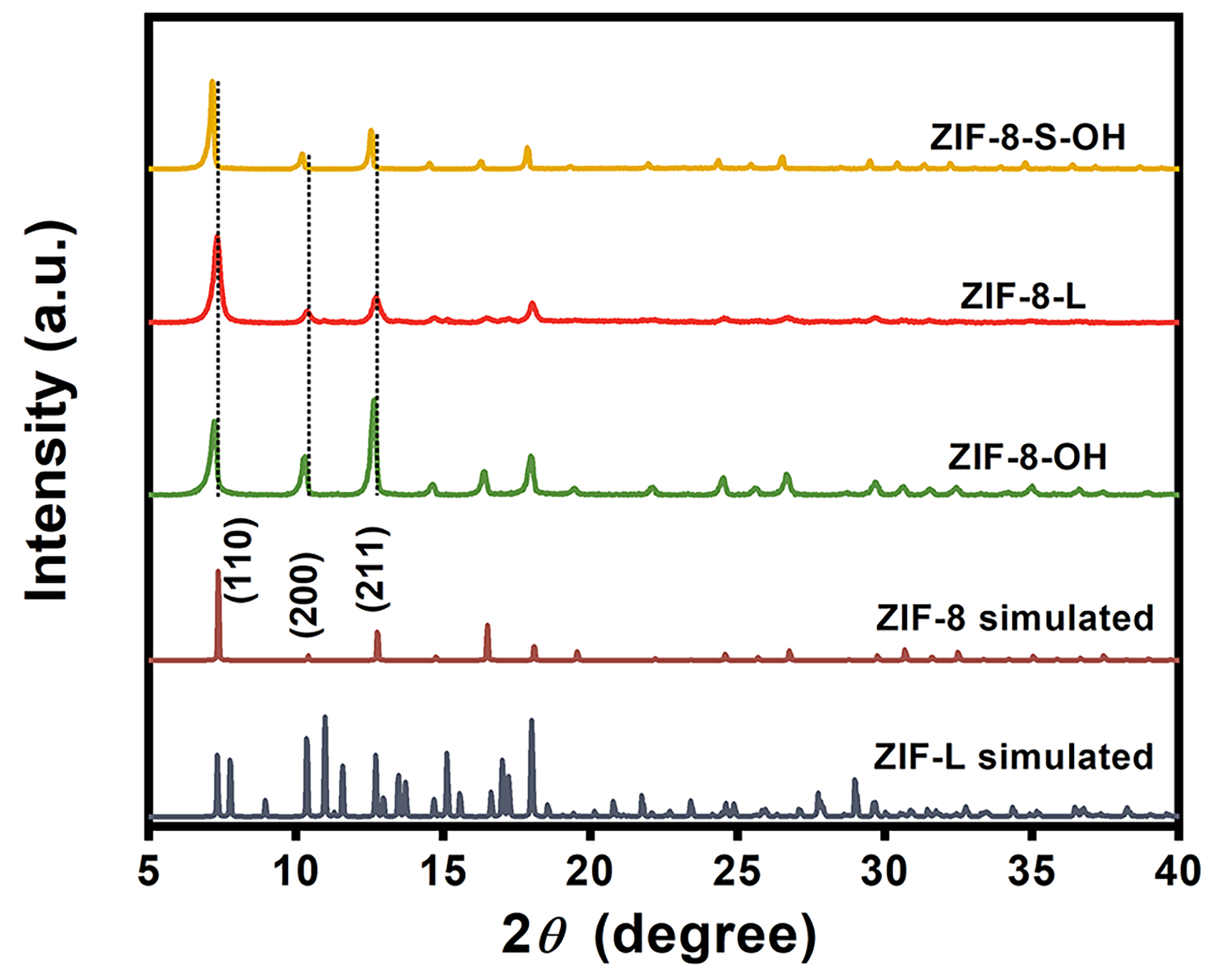

Supplement: Supplementary 1 — Figs. S1 to S16 [file research.0434.f1.zip › Fig. S4.tif]

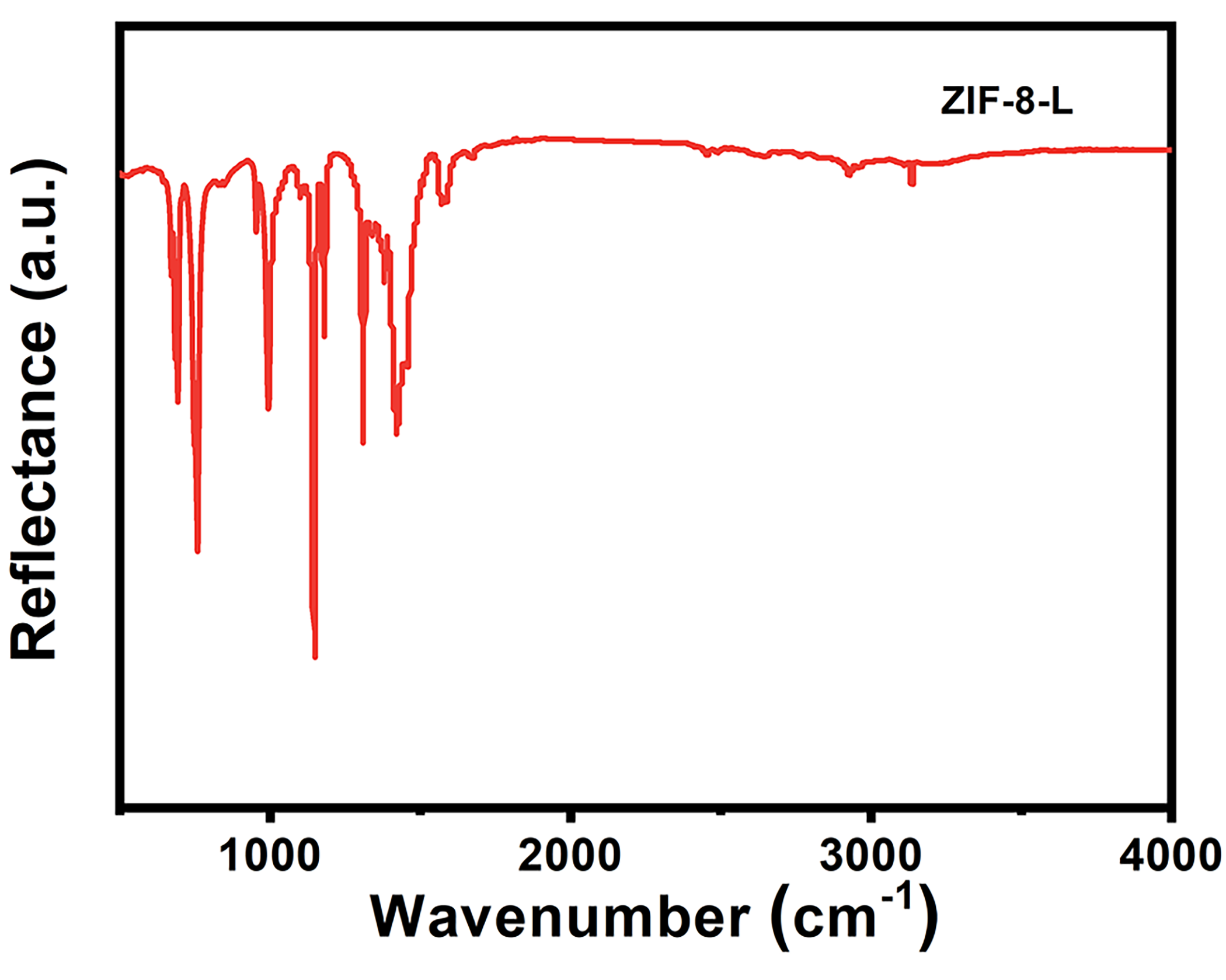

Supplement: Supplementary 1 — Figs. S1 to S16 [file research.0434.f1.zip › Fig. S5.tif]

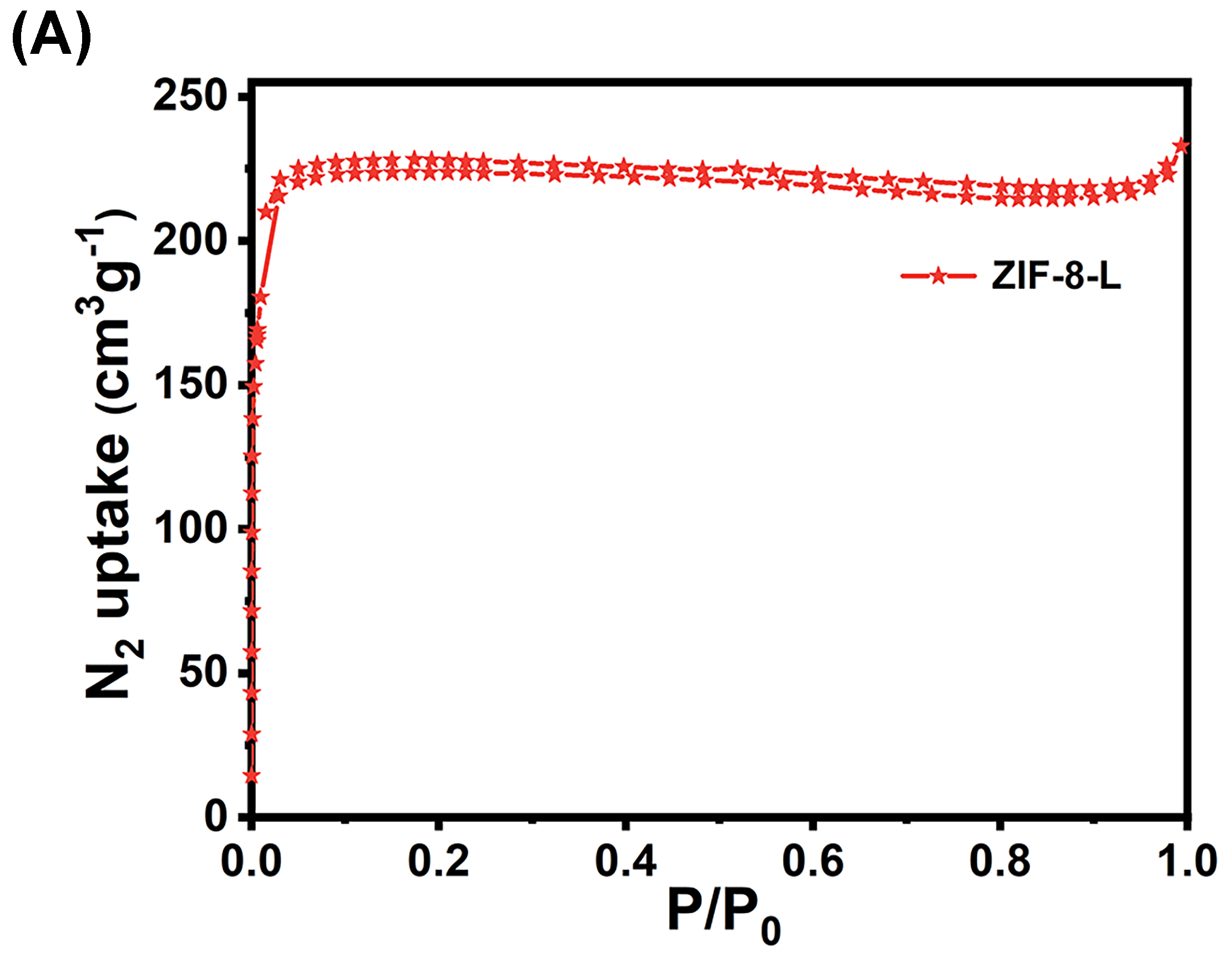

Supplement: Supplementary 1 — Figs. S1 to S16 [file research.0434.f1.zip › Fig. S6A.tif]

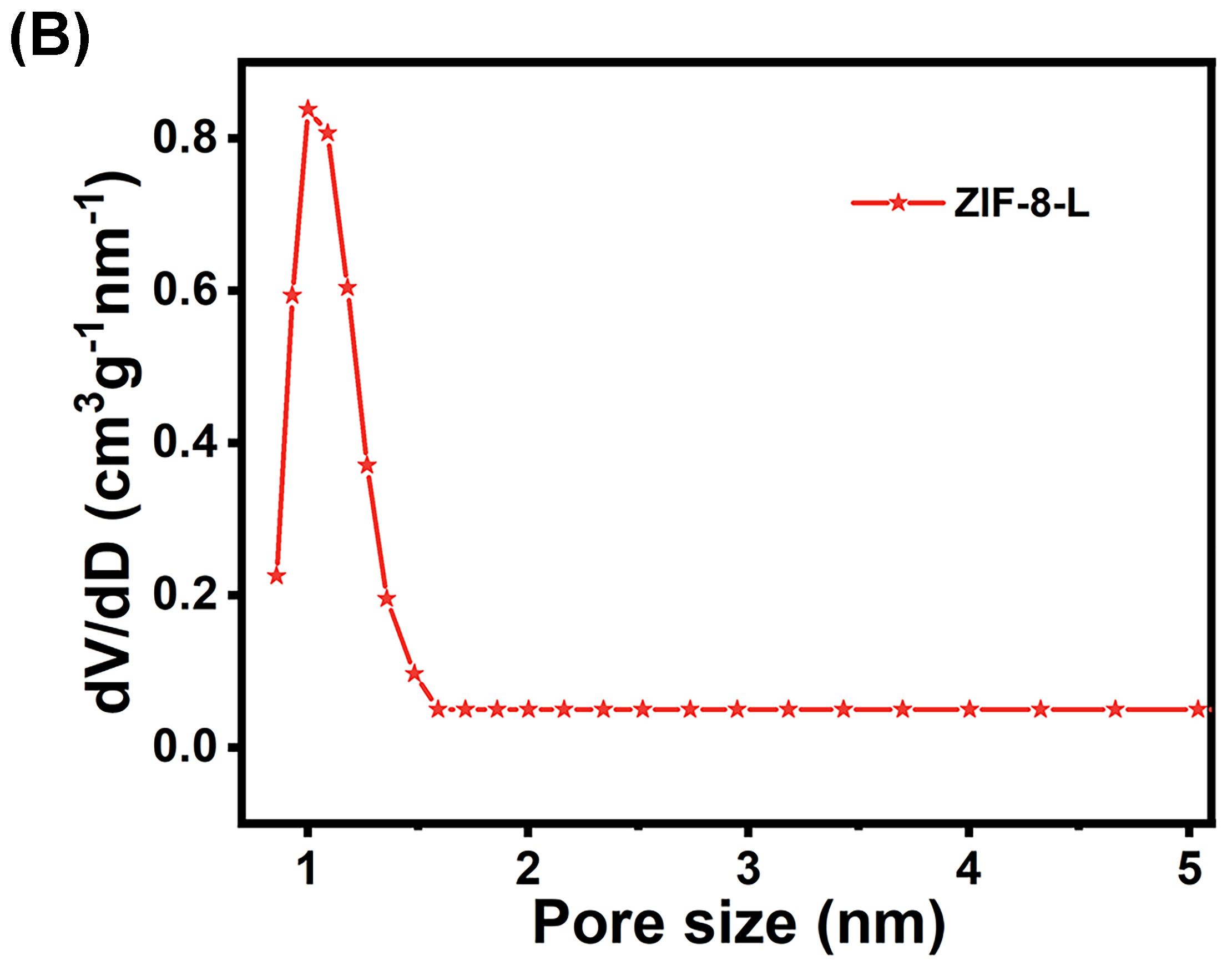

Supplement: Supplementary 1 — Figs. S1 to S16 [file research.0434.f1.zip › Fig. S6B.tif]

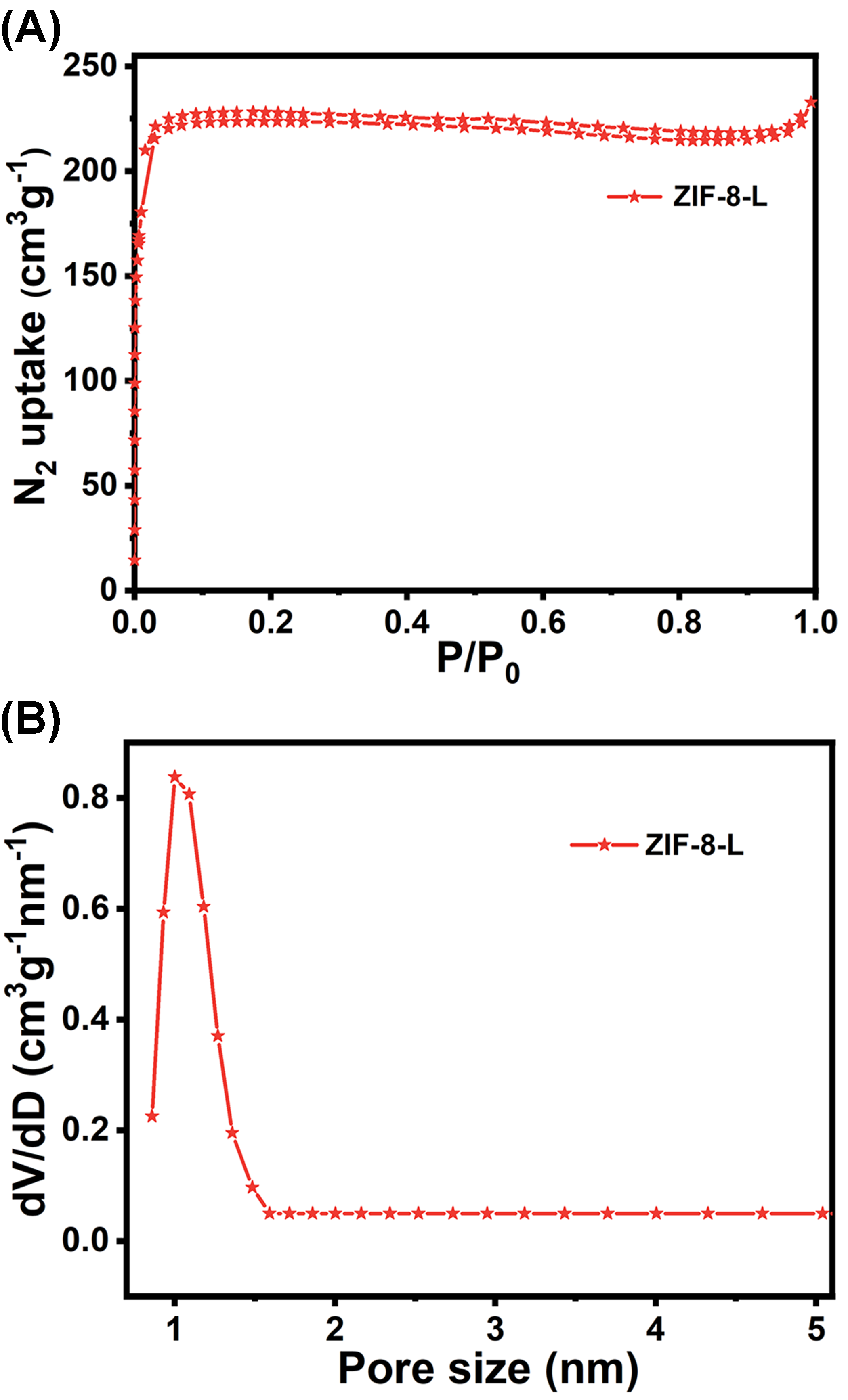

Supplement: Supplementary 1 — Figs. S1 to S16 [file research.0434.f1.zip › Fig. S6-contain ps layer.tif]

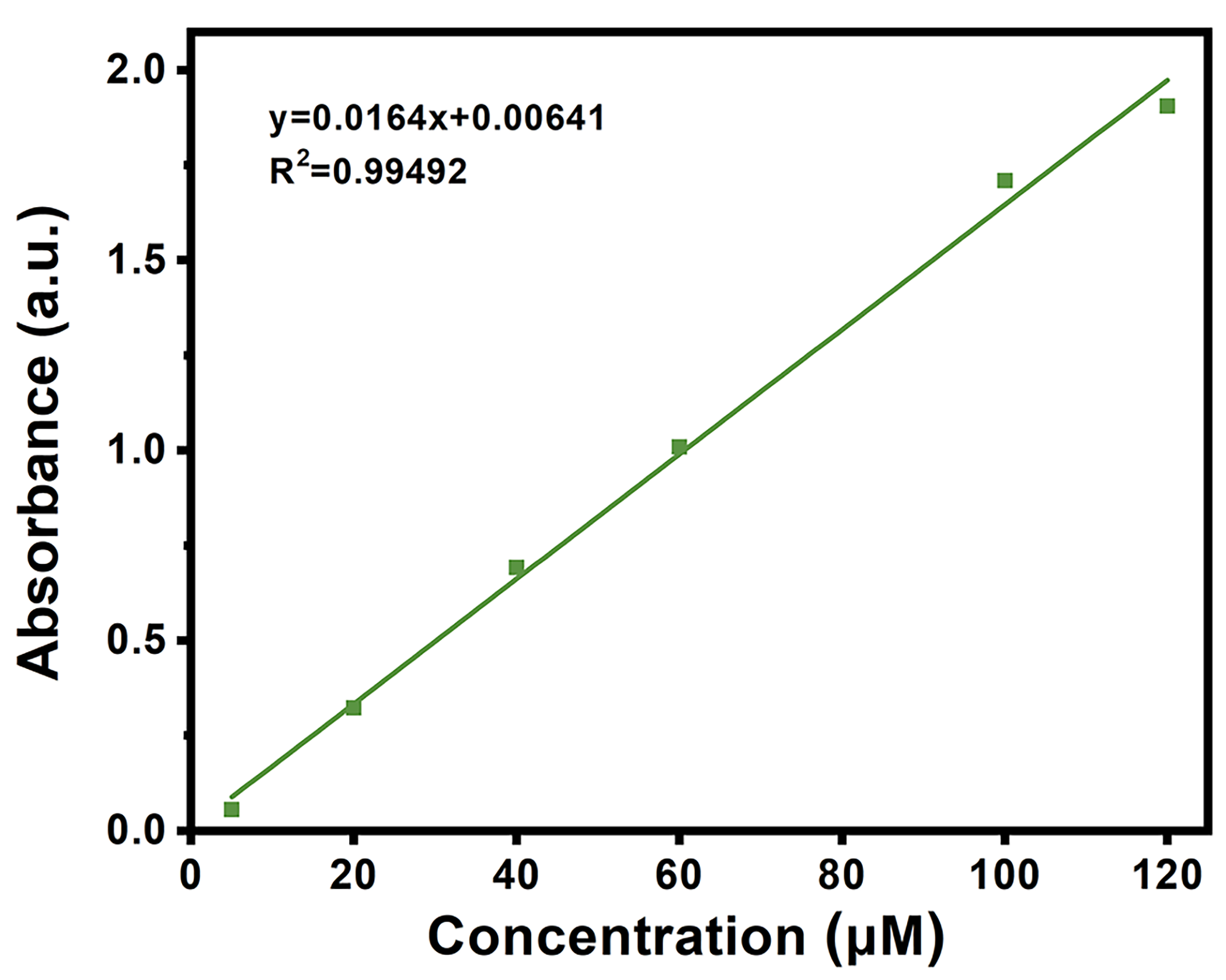

Supplement: Supplementary 1 — Figs. S1 to S16 [file research.0434.f1.zip › Fig. S7.tif]

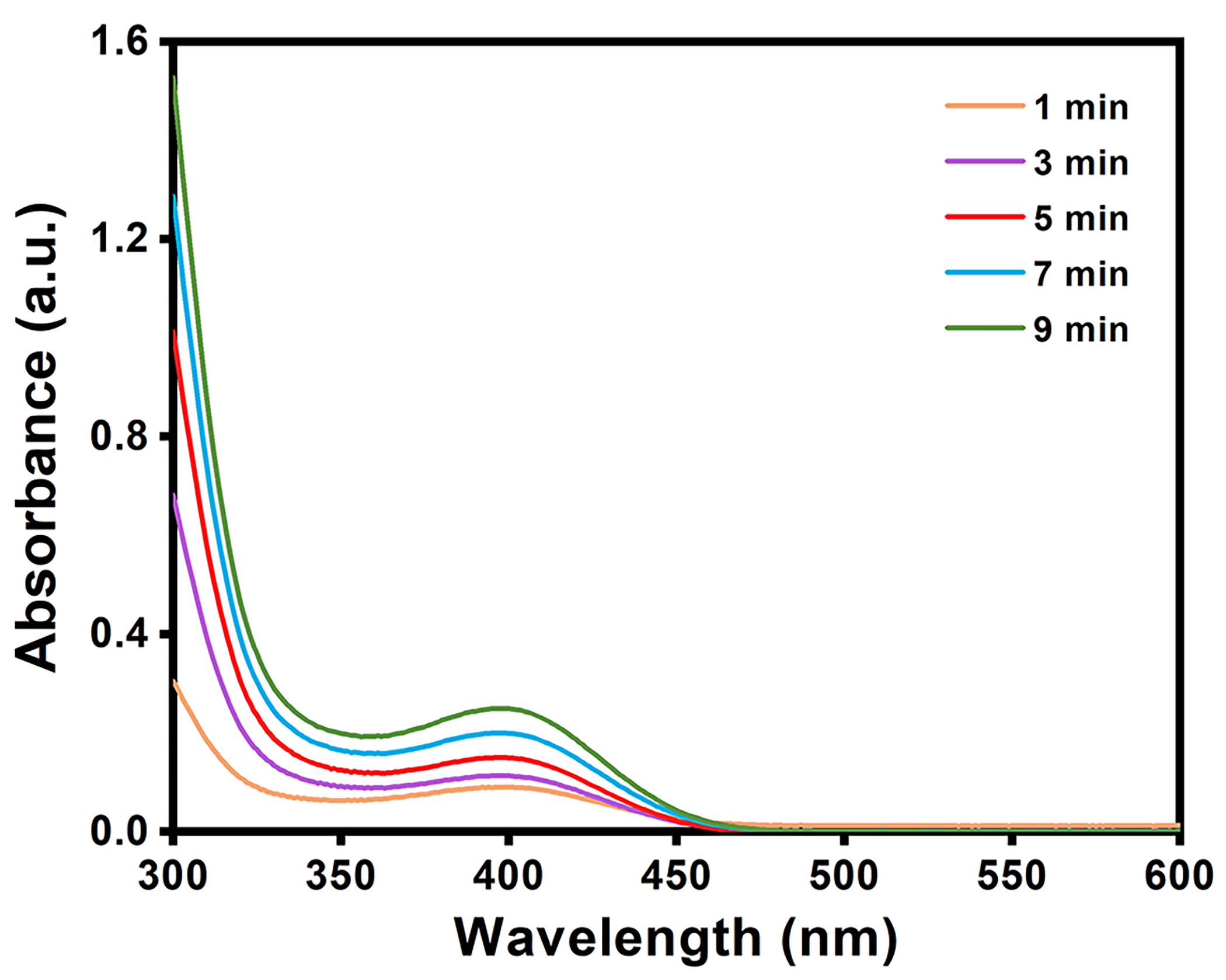

Supplement: Supplementary 1 — Figs. S1 to S16 [file research.0434.f1.zip › Fig. S8.tif]

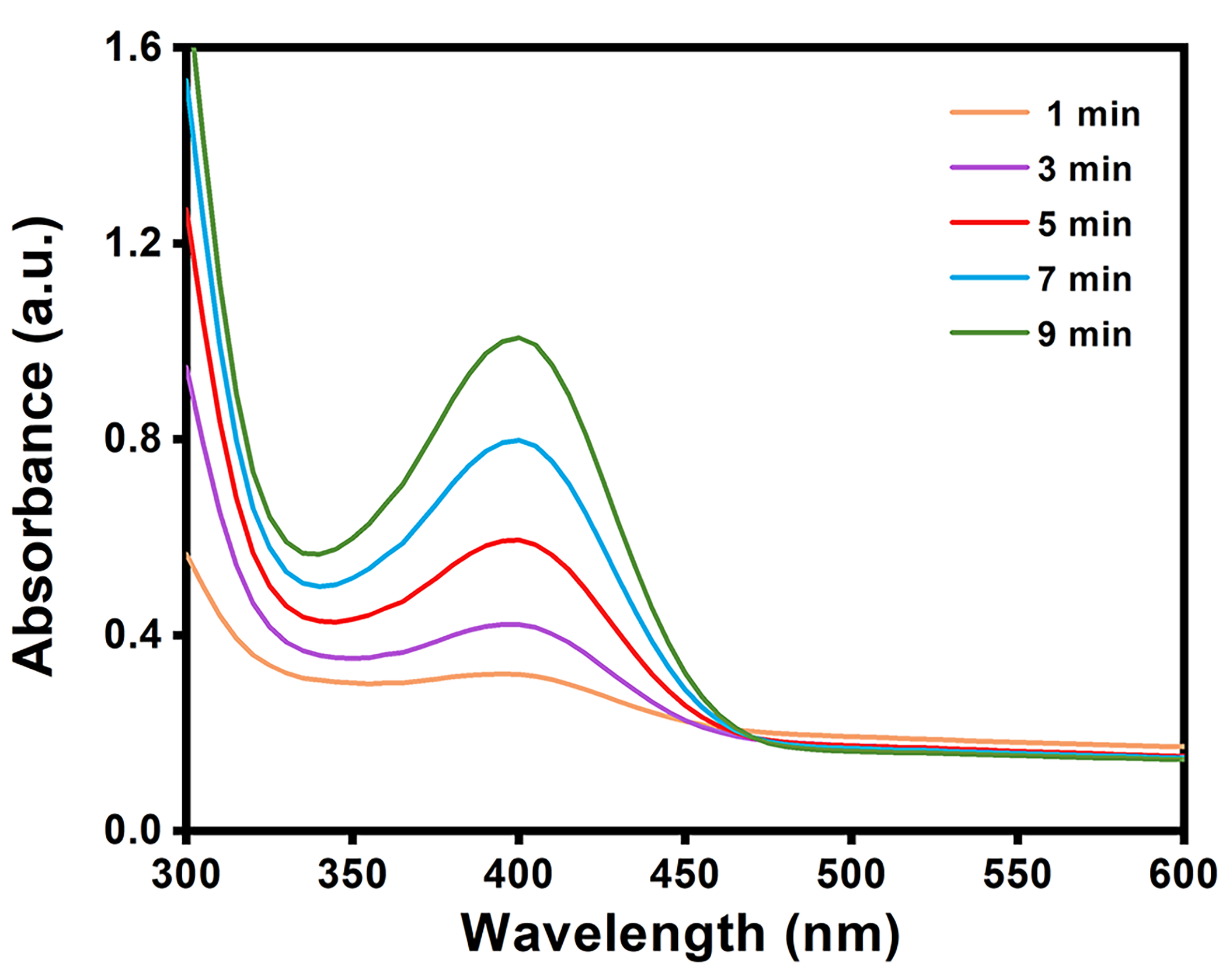

Supplement: Supplementary 1 — Figs. S1 to S16 [file research.0434.f1.zip › Fig. S9.tif]
